# A Game Theoretic Analysis of Competition Between Vaccine and Drug Companies during Disease Contraction and Recovery

**DOI:** 10.1177/0272989X211053563

**Published:** 2021-11-05

**Authors:** Kjell Hausken, Mthuli Ncube

**Affiliations:** Faculty of Science and Technology, University of Stavanger, Stavanger, Norway; Said Business School, University of Oxford, Oxford, UK

**Keywords:** COVID-19, death, disease contraction, donors, drug companies, drug development, game theory, patients, recovery, safe versus risky behavior, subsidies, vaccine companies, vaccine development

## Abstract

**Background:**

Infectious diseases such as COVID-19 and HIV/AIDS are behaviorally challenging for persons, vaccine and drug companies, and donors.

**Methods:**

In 3 linked games in which a disease may or may not be contracted, N persons choose risky or safe behavior (game 1). Two vaccine companies (game 2) and 2 drug companies (game 3) choose whether to develop vaccines and drugs. Each person chooses whether to buy 1 vaccine (if no disease contraction) or 1 drug (if disease contraction). A donor subsidizes vaccine and drug developments and purchases. Nature probabilistically chooses disease contraction, recovery versus death with and without each drug, and whether vaccines and drugs are developed successfully. COVID-19 data are used for parameter estimation.

**Results:**

Each person chooses risky behavior if its utility outweighs safe behavior, accounting for nature’s probability of disease contraction which depends on how many are vaccinated. Each person buys a vaccine or drug if the companies produce them and if their utilities (accounting for side effects and virus mutation) outweigh the costs, which may be subsidized by a sponsor.

**Discussion:**

Drug purchases depend on nature’s recovery probability exceeding the probability in the absence of a drug. Each company develops and produces a vaccine or drug if nature’s probability of successful development is high, if sufficiently many persons buy the vaccine or drug at a sales price that sufficiently exceeds the production price, and if the donor sponsors.

**Conclusion:**

Accounting for all players’ interlinked decisions allowing 14 outcomes, which is challenging without a game theoretic analysis, the donor maximizes all persons’ expected utilities at the societal level to adjust how persons’ purchases and the companies’ development and production are subsidized.

**Highlights:**

- A game theoretic approach can help explain the production decisions of vaccine and drug companies, and the decisions of persons and a donor, impacted by Nature.
- In 3 linked games, *N* persons choose risky behavior if its utility outweighs safe behavior.
- Vaccine and drug companies develop vaccines and drugs sponsored by a donor if profitable, allowing 14 outcomes.

## Introduction

### Background

Infectious diseases challenge humankind, with health, social, political, and economic consequences. As of January 16, 2021, 95 million were infected with COVID-19, and 2 million had died.^1^ In 2019, 38 million people lived with HIV/AIDS.^2^ Such diseases pose behavioral challenges for persons regarding attitudes toward risk; challenges for vaccine and drug companies regarding development, production, and sale; and challenges for societies represented, for example, by a donor that may subsidize. If too many persons choose risky behavior, if too few are vaccinated or if vaccines are unavailable, and if drugs are ineffective or unavailable, persons may die prematurely or recover with negative health effects, with societal impact.^3–5^

### Literature

#### Nongame theoretic studies

##### Prevention

Fitzpatrick et al.^6^ recommended the creation of a congressional cost-effectiveness committee to promote societal welfare and reveal underinvestment in public health compared with other sectors. If underinvestment is documented, donors are needed with the appropriate incentives. This article shows how a donor’s choices depend on the benefits to all persons of subsidization to induce vaccine and drug purchases, minus the subsidization costs, which may be adjusted to obtain optimal public health investment. Galárraga et al.^7^ argued that prevention programs are insufficiently implemented, causing more than 7000 HIV infections per day, due to unconvincing evidence of cost-effectiveness and challenges in comparing programs.^i^ Understanding the forces driving such a high number of infections relates to nature’s probability of disease contraction modeled in this article. This probability plays a role in the persons’ and donor’s expected utilities, which affect whether persons choose risky behavior, whether they buy vaccines and drugs, and whether the donor subsidizes, all of which in turn affect whether the companies develop, produce, and sell vaccines and drugs.

##### Prevention and treatment

To combat HIV/AIDS, Hogan et al.^15^ recommended mass media campaigns, interventions for sex workers, and treatment of sexually transmitted infections when resources are scarce, and when resources are not scarce, they recommended prevention of mother-to-child transmission, voluntary counseling and testing, and school-based education.^iv^ These recommendations are of paramount importance to inform persons, analyzed in this article, to understand the benefits and costs of risky and safe behavior, in particular the utilities of vaccination, recovery, and death, as well as nature’s choice, also analyzed in this article, of the probabilities of disease contraction with and without vaccination and the probabilities of recovery and death with and without drugs. Granich et al.^25^ found that increasing the provision of antiretroviral therapy (ART) to <350 cells/mm^3^ may decrease the HIV burden and associated costs. They estimated cost-effectiveness for the period 2011 to 2050. This finding is relevant for persons and donors, analyzed in this article, who have to pay and subsidize less and for vaccine and drug companies, also analyzed in this article, which may produce vaccines and drugs more cost-efficiently. Bärnighausen et al.^26^ contended that antiretroviral therapy costs and outcomes in current HIV TasP programs are unlikely to generalize to other TasP programs, recommending less detailed cost functions. Nevertheless, such costs and outcomes, whether they are quantified or not, play a role in the persons’ and donor’s expected utilities, and the vaccine and drug companies expected profits, as analyzed in this article, which suggests a need to assess these as proposed in this article.

##### Treatment

Forsythe et al.^27^ documented substantially improved health achievements and economic benefits and decreased costs during 20 y of ART. DiMasi et al.^28^ estimated $2.6 billion for HIV drug research and development costs for the years 2017 to 2021. West and Schneider^29^ estimated revenues for HIV/AIDS treatment for the years 2017 to 2021 for some African countries. Such costs are important inputs for the vaccine and drug companies’ expected profit functions in this article, affecting whether benefits outweigh costs and whether vaccines and drugs should be developed and produced. Kremer and Snyder,^30,31^ Thomas,^32^ and Kremer and Glennerster^33^ found that incentives for developing treatment drugs are stronger than incentives for developing prevention vaccines. Hence, more citizens may become sick, causing countries with a high disease prevalence to allocate more resources to treatment than to prevention. This finding is particularly relevant for the donor’s decision making in this article. In particular, the donor may subsidize development, production, and purchases (all 3 of which are analyzed in this article) of vaccines more than drugs, thus favoring prevention more than treatment.

#### Game theoretic studies

Game theoretic contributions are rare for this phenomenon. Hausken and Ncube^34^ considered 5 outcomes in a game between persons and 1 pharmaceutical company (i.e., safe behavior, risky behavior and no disease contraction, disease contraction without drug availability, disease contraction with drug availability but without buying the drug, and disease contraction and buying the drug). They illustrated with HIV/AIDS data how a parametric donor affects drug development and drug purchases. Mamani et al.^35^ recommended a contractual mechanism to remedy the inefficient allocation of influenza vaccines within multiple countries affected by the interdependent risk of infection across borders. They demonstrate decreased global costs of infection and fewer infections, especially with high cross-border transmission rates. Hausken and Ncube^36,37^ assessed policy makers choosing resource allocation between disease prevention and treatment, the international community choosing disease treatment funding, and nature choosing population fractions of disease contraction, sickness/death, and recovery. They illustrated free riding and found that more resource allocation for prevention causes less disease contraction but a higher death rate given disease contraction. This article contributes to this literature by modeling more players than in the above studies do (i.e., persons, vaccine companies, drug companies, a donor, and nature), accounting for more relationships between the players and better explaining how the games relate to the vaccine and drug companies’ research and development, production, bringing their products to the market, and sale.

### Contribution

Health policy decisions are not usually analyzed game theoretically. This article incorporates the relevant players game theoretically. Game theory requires at least 2 players, with at least 1 player choosing at least 2 strategies and each player receiving a payoff given the combinations of strategies chosen by all players. Enabling each player’s strategy to depend mutually on all the players’ strategies allows for a different kind of analysis compared with when each player chooses a strategy or makes a decision unilaterally considering the environment as fixed or immutable.

The research question is to determine how each player weighs which benefits against which costs when choosing between which strategies. That question is particularly relevant for this phenomenon, where life and death depend on all the players’ interlinked decisions. More specifically, each person chooses between safe behavior, which may preserve life but may be more restrictive and be less exciting than risky behavior, which may cause disease. Each person also chooses whether or not to buy 1 vaccine if the disease is not contracted and 1 drug if the disease is contracted. That choice depends on the vaccine or drug being available at acceptable prices; which depends on donor subsidies; the utility of vaccination (which may have side effects or may not handle virus mutation); the utilities of recovery, death, and risky versus safe behavior; and nature’s probabilities of disease contraction and recovery with and without a drug.

Two vaccine companies and 2 drug (pharmaceutical) companies choose whether or not to develop and produce vaccines and drugs. These choices depend on the development costs affected by sponsor subsidies and nature’s probabilities of whether the development succeeds, on the production costs and sales prices, and on how many persons buy vaccines and drugs. A donor chooses subsidization of vaccine and drug development and vaccine and drug purchases for persons. The donor’s choices depend on all persons’ benefits of subsidization to induce vaccine and drug purchases, weighed against the subsidization costs. Nature chooses the disease contraction probability given risky behavior, the disease recovery probability without drugs, the disease recovery probabilities with each of the 2 drugs, and whether vaccines and drugs are developed successfully. This conceptualization is intended to better address the challenges in designing a strategic response to a pandemic or epidemic infectious disease.

This article contributes to the prevention literature by applying game theory to model whether and how vaccine companies develop vaccines and how persons buy vaccines, sponsored by a donor, and affected by nature choosing probabilities of disease contraction, recovery and death, and whether vaccines and drugs are developed successfully. This article contributes to the treatment literature by applying game theory to model whether and how drug companies develop drugs and how persons buy drugs, sponsored by a donor, and affected by nature choosing probabilities of disease contraction, recovery and death, and whether vaccines and drugs are developed successfully.

### Article Organization

The “Methods” section presents the methods, design, and model. The “Results” section provides the results. The “Discussion” section discusses the results, with limitations, future research, and literature review. The last section concludes.

## Methods

### Overview

The subject of study is potential disease contraction depending on persons choosing risky or safe behavior, potential vaccine and drug developments by vaccine and drug companies, potential purchases of vaccines and drugs by persons, potential subsidization by a donor, and potential recovery or death (or decreased life quality) for persons contracting the disease.

N persons, 2 vaccine companies, 2 drug companies, and the donor play 3 linked games impacted by nature. The games are solved with backward induction. Nature chooses recovery or death probabilistically and whether vaccines and drugs are developed successfully. Each person buys 1 of 2 vaccines or no vaccines if not contracting the disease and 1 of 2 drugs or no drugs if contracting the disease, subsidized by a donor. The vaccine and drug companies develop or do not develop vaccines and drugs sponsored by a donor. Nature chooses the disease contraction probabilistically. The N persons choose risky or safe behavior. The research questions are which strategies the N persons, 2 vaccine companies, 2 drug companies, and donor choose and which of 14 outcomes follow. Outcome 1 follows from safe behavior. Outcome 2 follows from risky behavior without disease contraction and vaccination. Outcomes 3 to 6 follow from risky behavior, no disease contraction, and vaccination. Outcomes 7 to 14 follow from risky behavior and disease contraction, which causes recovery or death.

The model applies to diseases satisfying 3 criteria. First, disease contraction depends on each person choosing risky or safe behavior (e.g., not wearing a mask, washing hands, and keeping distance against COVID-19 or not using a condom or avoiding multiple partners against HIV, hence excluding genetically predisposed and behaviorally independent diseases). Second, vaccine and drug companies are assumed that may develop vaccines and drugs. If vaccines or drugs cannot be produced for the given disease, the model simplifies to the special case when vaccines or drugs are unavailable. Third, we assume diseases enabling recovery in various degrees with or without drugs.

The model is illustrated and parameters estimated with early COVID-19 data on the BioNTech/Pfizer Comirnaty vaccine and the Moderna vaccine and the experimental drugs hydroxychloroquine and ivermectin. These 2 controversial off-the-shelf drugs were chosen because they currently have available prices. The model applies equally well for drugs involving, for example, new molecular entities developed over many years if prices and development costs can be estimated.

We show how the players strike balances when making their decisions in a game theoretic cost-benefit analysis. Each person may buy 1 drug given disease contraction and 1 vaccine otherwise. The vaccine and drug companies may or may not enable such purchases. The donor may or not sponsor. Nature may choose disease contraction, recovery and death, and whether vaccines and drugs are developed successfully, in multifarious ways.

### Conceptualization

**Figure 1:**
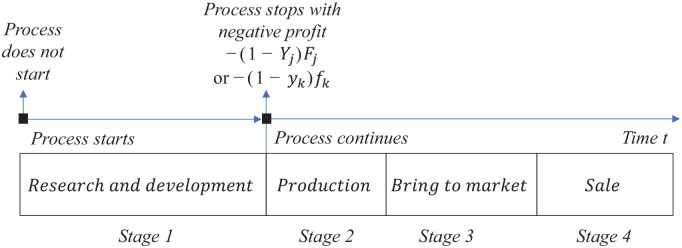
Vaccine company k ’s and drug company j ’s timeline of research and development, production, bringing its product to the market, and sale, j,k=1,2 . Decision nodes by each company are squares.

Figure 1 shows vaccine company k ’s, k=1,2 , and drug company j ’s, j=1,2 , timeline of research and development, production, bringing its product to the market, and sale. Supplementary Appendix A shows the nomenclature. Each company chooses before stage 1 either to start the research and development process or not to start the process, earning zero profit. If the process starts, each company chooses before stage 2 either to continue with production, bringing its product to the market, and sale, or to stop the process earning negative profit $-\left(1-Y_{j}\right)F_{j}$ for drug company j , and negative profit $-\left(1-y_{k}\right)f_{k}$ for vaccine company k,j,k=1,2 . If the health authorities do not approve the vaccine or drug, that may be interpreted so that the vaccine or drug does not get produced.

**Figure 2:**
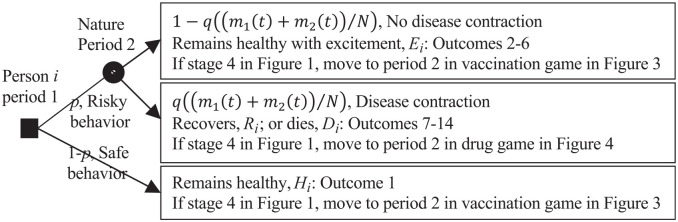
Two-period game between person *i*, *i* = 1, . . ., *N*, in period 1 and nature in period 2 on safe versus risky behavior and whether or not to contract the disease. The filled square decision node is for person *i*. The filled circle chance node is for nature.

**Figure 3:**
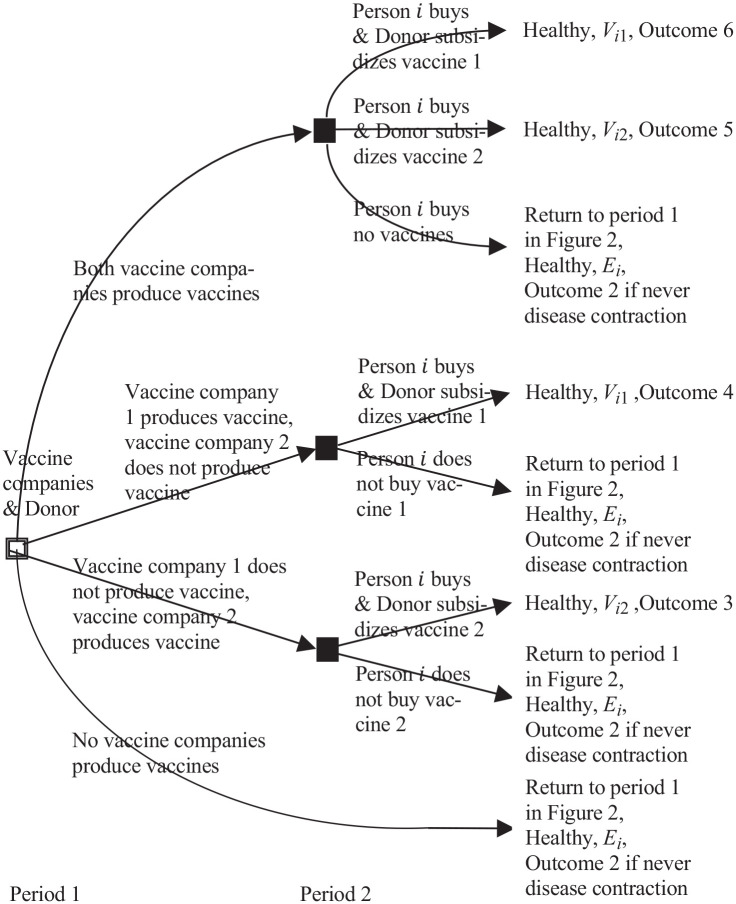
Two-period game between vaccine companies 1 and 2, the donor, and the part of *N* persons who have not contracted the disease. The framed square decision node (unfilled square with 2 demarcating lines along each side) is for the vaccine companies and the donor. The filled square decision nodes are for person *i*.

**Figure 4:**
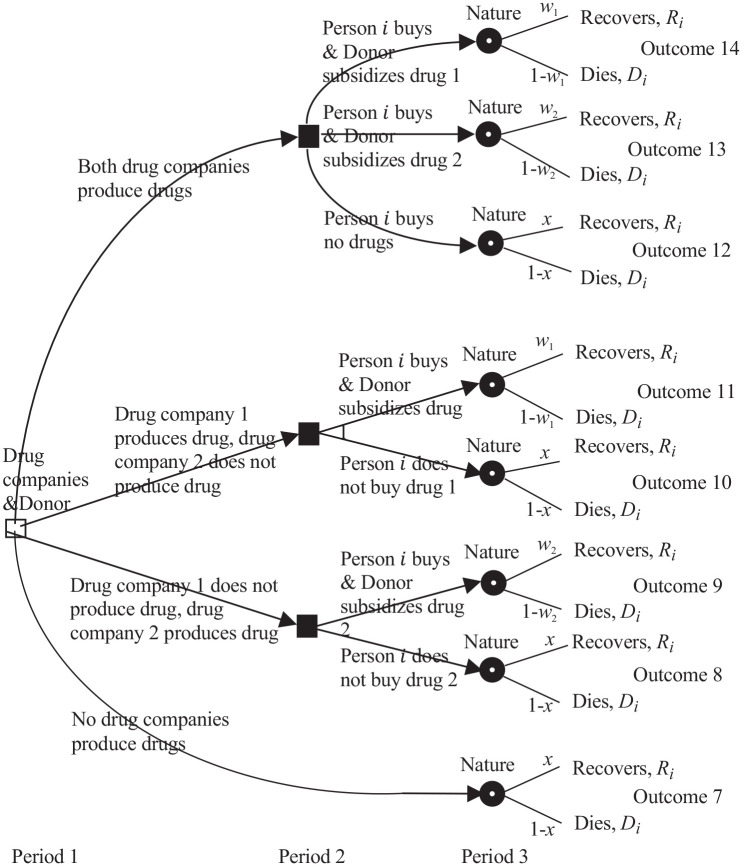
Three-period game between drug companies 1 and 2, the donor, the part of the persons *N* who have contracted the disease, and nature. The unfilled square decision node is for the drug companies and the donor. The filled square decision nodes are for person *i*. The filled circle chance *i* nodes are for nature.

The players, that is, N persons, 2 vaccine companies, 2 drug companies, and the donor play 3 linked games affected by nature, as described in Figures 2 through 4. All players are assumed to be rational and maximize conventional expected utility theory. Figure 2 shows the complete information 2-period game between person i , i=1,…,N , who chooses risky or safe behavior in period 1, and nature, which chooses whether or not the disease is contracted in period 2. Period 2 starts immediately after person i has made its choice in period 1. A fraction p , determined as a consequence of the N persons’ choices, and hence pN persons, chooses risky behavior. Risky behavior causes disease contraction chosen by nature with probability $q\left(\left(m_{1}\left(t\right)+m_{2}\left(t\right)\right)/N\right)$ in period 2, where $\left(m_{1}\left(t\right)+m_{2}\left(t\right)\right)/N$ is the fraction of the N persons who have been vaccinated at time t and $m_{k}\left(t\right)$ is the number of persons having bought and been vaccinated by vaccine k , k=1,2 , at time t,t≥0 . The disease contraction probability $q\left(\left(m_{1}\left(t\right)+m_{2}\left(t\right)\right)/N\right)$ decreases as the fraction of the N persons who has been vaccinated at time t increases, that is, $\partial q\left(\left(m_{1}\left(t\right)+m_{2}\left(t\right)\right)/N\right)/\partial \left(\left(m_{1}\left(t\right)+m_{2}\left(t\right)\right)/N\right)\leq 0$ . Thus, $\mathrm{pq}\left(\left(m_{1}\left(t\right)+m_{2}\left(t\right)\right)/N\right)N$ persons have contracted the disease at time t . The game in Figure 2 is played within all 4 stages in Figure 1. If stage 4 in Figure 1 is reached, person i moves to period 2 in the vaccination game in Figure 3 if the disease is not contracted and moves to period 2 in the drug game in Figure 4 if the disease is contracted.

Figure 3 shows the 2-period complete information game between vaccine companies 1 and 2, the donor,^iii^ and the N persons. In period 1, assuming that the disease is not contracted, vaccine company k chooses whether to produce vaccine k , k=1,2 , and the donor chooses sponsoring. In period 2, person i chooses whether or not to buy 1 vaccine, when and if it is available, and the donor chooses sponsoring.

Figure 4 shows the 3-period game between drug companies 1 and 2, the donor, the N persons, and nature. In period 1, assuming that the disease is contracted, drug company j chooses whether to produce drug j , j=1,2 , and the donor chooses sponsoring. In period 2, person i chooses whether or not to buy 1 drug, when and if it is available, and the donor chooses sponsoring. In period 3, nature chooses recovery versus death. The recovery and death processes depend on the disease and person i . For COVID-19 and HIV/AIDS, these processes usually take months. Supplementary Appendix B describes the games more extensively, with analysis in Supplementary Appendix C and the 14 outcomes in Supplementary Appendix D.

## Results

### Overview

**Figure 5:**
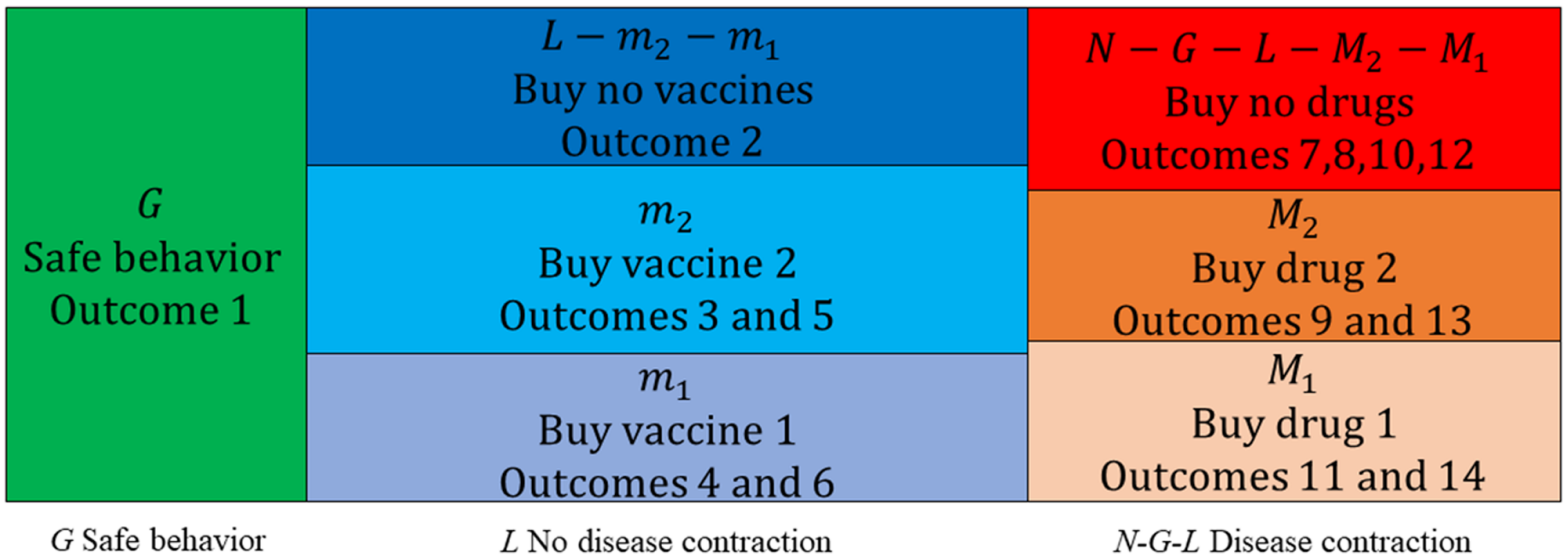
*N* persons dispersed across 7 groups.

Person i ’s 14 outcomes in Figure 2, Figure 3, and Figure 4 cause the N persons to disperse across the 7 groups in Figure 5. The 7 groups are the G persons choosing safe behavior; the L persons choosing risky behavior while not contracting the disease, which are split into 3 groups ( $L-m_{2}-m_{1}$ buying no vaccines, $m_{2}$ buying vaccine 2, and $m_{1}$ buying vaccine 1); and the N−G−L persons choosing risky behavior while contracting the disease, which are also split into 3 groups ( $N-G-L-M_{2}-M_{1}$ buying no vaccines, $M_{2}$ buying vaccine 2, and $M_{1}$ buying vaccine 1), where $m_{k}=\underset{t\to \infty }{\lim}m_{k}\left(t\right)$ .

### Numerical Example Estimating the Parameters

#### Benchmark scenario with outcomes 5, 6, and 14

This section estimates the model’s parameters; the donor’s 8 strategic choices $y_{k}$ , $s_{k}$ , $Y_{j}$ , $S_{j}$ ; and nature’s 8 strategic choices, $q\left(\left(m_{1}\left(t\right)+m_{2}\left(t\right)\right)/N\right)$ , x , $w_{j}$ , $g_{\mathrm{vk}}$ , $g_{\mathrm{dj}}$ , j,k=1,2 . We consider the two COVID-19 vaccines Comirnaty from BioNTech/Pfizer and Moderna from Moderna, which early completed phase 3 testing. On November 23, 2020, BioNTech/Pfizer announced a price of $20 for 1 dose of the Comirnaty vaccine, whereas Moderna announced a price of $10 to $50 for 1 dose of the Moderna vaccine, depending on the amount ordered.^39^ Hence, we set the vaccine k purchasing cost for person i at $c_{k}=\$20,k=1,2$ .^iv^ Also, here we assume that the vaccine k production cost $b_{k}$ for vaccine company k destined for person i is 25% of the vaccine k purchasing cost $c_{k}$ , that is, $b_{k}=\$5,k=1,2$ . To reflect efficient production and large markets, we set the exponential parameter $a_{k}$ , which scales the vaccine k production cost equal to $a_{k}=0.5$ , k=1,2 . As for the drug j development costs $F_{j}$ for hydroxychloroquine and ivermectin, we assume the same ratio $2.6 billion/$100 for the vaccine k development costs of the Comirnaty vaccine from BioNTech/Pfizer and the Moderna vaccine from Moderna, that is, $f_{k}=\$2.6\times 5/100=\$0.13$ billion ,k=1,2 . We set n=3 as the number of returns from period 2 in Figure 3 to period 1 in Figure 2 when assessing $E_{i}$ , with scaling parameter r=1 . We assume that $m_{k}=20$ million persons buy vaccine k , k=1,2 .

At the time of writing, few drugs against COVID-19 exist. We thus consider the 2 experimental and controversial drugs hydroxychloroquine and ivermectin, which have been used by some, although not approved by sufficiently many authorities. Lansdowne states that design, development and drug approval can take 10–15 years, or less if the drug is believed to outcompete existing drugs or is the only available treatment.^41^ One hundred tablets, each weighing 200 mg, of hydroxychloroquine cost $37.22.^42^ Consuming 400 mg per week for 1 y^43^ gives a drug 1 purchasing cost for 1 person of $C_{1}=\$38.71$ for 1 y. Twenty tablets each weighing 3 mg of ivermectin cost $79.07.^44^ Consuming 12.4 mg per week for 1 y,^45^ assuming 0.2 mg/kg for an average person weighing 62 kg, gives a drug 2 purchasing cost for 1 person of $C_{2}=\$849.74$ for 1 y. The drug production cost $B_{j}$ for drug company j destined for person i , i=1,…,N , is lower than $C_{j}$ . We choose 25% of the price, which gives $B_{1}=\$9.68$ and $B_{2}=\$212.44$ per person per year. The exponential parameter $A_{j}$ , which scales the drug j production cost, depends on the economy of scale. To reflect efficient production and large markets, we assume $A_{j}=0.5$ , j=1,2 . The drug j development costs $F_{j}$ for hydroxychloroquine and ivermectin are unknown. Hausken and Ncube^34^ assumed an HIV development cost of $2.6 billion, matched against a drug-purchasing cost for 1 person of $100 per year. Hypothetically assuming the same ratio of $2.6 billion/$100 for hydroxychloroquine and ivermectin gives the drug j development costs $F_{1}=\$2.6\times 9.68/100=\$0.25$ billion and $F_{2}=\$2.6\times 212.44/100=\$5.52$ billion. We assume that $M_{j}=20$ million persons buy drug j,j=1,2 .

Person i has 5 specific utilities: $E_{i}$ , $V_{\mathrm{ik}}$ , $H_{i}$ , $R_{i}$ , $D_{i}$ , i=1,…,N . Appelbaum^46^ estimated the value of statistical life as $6.1 to $9.1 million.^v^ We thus choose $D_{i}=-\$7$ million, which expresses a strong negative value of death. We estimate person i ’s value of risky behavior as $E_{i}=\$1$ million, which is 1/7 of the value of statistical life, acknowledging that life consists of more than risky behavior. Nonrisky behavior also gives utility. Person i ^’s^ utility $V_{\mathrm{ik}}$ of vaccine k vaccination is lower than $E_{i}$ for risky behavior, $V_{\mathrm{ik}}<E_{i}$ , since vaccination may have uncertain side effects, may require time and effort, and so forth. We thus set $V_{\mathrm{ik}}=\$0.8$ million, k=1,2 . We set n=3 as the number of returns from period 2 in Figure 3 to period 1 in Figure 2 when assessing $E_{i}$ , with scaling parameter r=1 . We assume that person i ’s utility $H_{i}$ of safe behavior is lower than both $E_{i}$ for risky behavior and $V_{\mathrm{ik}}$ for vaccination, $H_{i}<V_{\mathrm{ik}}$ , since vaccination is not needed if person *i* is guaranteed to choose safe behavior, which can be quite restrictive for a disease such as COVID-19. We thus set $H_{i}=\$0.5$ million. Person i ’s utility $R_{i}$ when recovering from the disease is even lower than $H_{i}$ for safe behavior, $R_{i}<H_{i}$ . We thus set $R_{i}=\$0.2$ million.

The donor’s 8 subsidy fractions are currently unknown. We estimate $Y_{j}=S_{j}=y_{k}=s_{k}=0.5$ , j,k=1,2 , which means that the donor subsidizes 50%.

To estimate nature’s 8 strategic choices, observe that 116 million people have contracted COVID-19 by March 5, 2021, 65.5 million have recovered, and 2.6 million have died,^47^ out of the world’s 7.85 billion population.^48^ Dividing 116 million by 7.85 billion gives the fraction 0.01478. If we hypothetically assume that 785 million will have contracted COVID-19 at some time in the future, we get the disease contraction probability $q\left(\left(m_{1}\left(t\right)+m_{2}\left(t\right)\right)/N\right)=0.1$ . We hypothetically assume the higher disease contraction probability q(0)=0.3 if no one buys vaccines, that is, $m_{1}\left(t\right)=m_{2}\left(t\right)=0$ . Dividing 65.5 million by 116 million for disease recovery gives the fraction 0.5647. Dividing 116-2.6 = 113.4 million by 116 million for avoiding death by March 5, 2021, given disease contraction at any point in time, gives the fraction 0.9776. We estimate the disease recovery probability x=0.9 without drug j and disease recovery probability $w_{j}=0.95$ with drug j , j=1,2 . We consider both $g_{\mathrm{vk}}=0=g_{\mathrm{dj}}$ and $g_{\mathrm{vk}}=1=g_{\mathrm{dj}}$ , which are the probabilities for whether vaccine k and drug j are developed successfully.

Applying the above parameter values gives the results in the 2 rightmost columns in Table 1 (Supplementary Appendix D). Using the game tree in Figures 2–4, outcome 1 is impossible and person i chooses risky behavior, which changes if person i ’s utility $H_{i}$ of safe behavior increases sufficiently above $H_{i}=\$0.5$ million. If disease contraction occurs, person i focuses on drugs. Person i chooses outcome 14, which means buying the cheapest drug 1, where both drug companies develop drugs. If drug 2 is cheaper, person i chooses outcome 13 instead. If both drugs are too expensive, person i chooses outcome 12. If drug 1 or drug 2 is produced, which person i may buy or not buy, person i chooses one of the outcomes 8, 9, 10, or 11. If no drugs are produced, person i chooses outcome 7. If disease contraction does not occur, person i focuses on vaccines. Person i chooses either outcome 5 or outcome 6, but not both, which means buying either vaccine 1 or vaccine 2, which are equally expensive. If both vaccines are too expensive, person i chooses outcome 2. If vaccine 1 or vaccine 2 is produced, which person i may buy or not buy, person i chooses one of the outcomes 2, 3, or 4. If no vaccines are produced, person i chooses outcome 2.

Inserting the parameter values into (9), person i ’s expected utility is

(1) $$W_{i}=\left\{ \begin{array}{c} \$500000\;\mathrm{if}\;\mathrm{safe}\;\mathrm{behavior}\;\&\;\mathrm{no}\;\mathrm{disease}\;\mathrm{contraction}\;\&\;\mathrm{no}\;\mathrm{vaccination}\\ \$729000\;\mathrm{if}\;\mathrm{risky}\;\mathrm{behavior}\;\&\;\mathrm{no}\;\mathrm{disease}\;\mathrm{contraction}\;\&\;\mathrm{no}\;\mathrm{vaccination}\\ \$799990\;\mathrm{if}\;\mathrm{rb}\;\&\;\mathrm{vaccine}\;2\;\mathrm{development}\;\&\;\mathrm{vaccination}\\ \$799990\;\mathrm{if}\;\mathrm{rb}\;\&\;\mathrm{vaccine}\;1\;\mathrm{development}\;\&\;\mathrm{vaccination}\\ \$799990\;\mathrm{if}\;\mathrm{rb}\;\&\;\mathrm{vaccines}\;1\;\&\;2\;\mathrm{develop}\;\&\;\mathrm{vaccine}\;2\;\mathrm{vaccination}\\ \$799990\;\mathrm{if}\;\mathrm{rb}\;\&\;\mathrm{vaccines}\;1\;\&\;2\;\mathrm{develop}\;\&\;\mathrm{vaccine}\;1\;\mathrm{vaccination}\\ -\$520000\;\mathrm{if}\;\mathrm{risky}\;\mathrm{beh}\;\&\;\mathrm{disease}\;\mathrm{contr}\;\&\;\mathrm{no}\;\mathrm{drug}\;\mathrm{development}\\ -\$520000\;\mathrm{if}\;\mathrm{risky}\;\mathrm{beh}\;\&\;\mathrm{dis}\;\mathrm{contr}\;\&\;\mathrm{drug}\;\mathrm{dev}\;\&\;\mathrm{not}\;\mathrm{buy}\;\mathrm{drug}\;2\\ -\$160425\;\mathrm{if}\;\mathrm{rb}\;\&\;\mathrm{dis}\;\mathrm{contr}\;\&\;\mathrm{dr}\;\mathrm{dev}\;\&\;\mathrm{buy}\;\mathrm{drug}\;2\\ -\$520000\;\mathrm{if}\;\mathrm{rb}\;\&\;\mathrm{dis}\;\mathrm{contr}\;\&\;\mathrm{drug}\;\mathrm{dev}\;\&\;\mathrm{not}\;\mathrm{buy}\;\mathrm{drug}\;1\\ -\$160019\;\mathrm{if}\;\mathrm{rb}\;\&\;\mathrm{dis}\;\mathrm{contr}\;\&\;\mathrm{drug}\;\mathrm{dev}\;\&\;\mathrm{buy}\;\mathrm{dr}\;1\\ -\$520000\;\mathrm{if}\;\mathrm{rb}\;\&\;\mathrm{dis}\;\mathrm{contr}\;\&\;\mathrm{drug}\;\mathrm{dev}\;\&\;\mathrm{not}\;\mathrm{buy}\;\mathrm{drugs}\;1\;\mathrm{or}\;2\\ -\$160425\;\mathrm{if}\;\mathrm{rb}\;\&\;\mathrm{dis}\;\mathrm{contr}\;\&\;\mathrm{drug}\;\mathrm{dev}\;\&\;\mathrm{buy}\;\mathrm{dr}\;2\\ -\$160019\;\mathrm{if}\;\mathrm{rb}\;\&\;\mathrm{dis}\;\mathrm{contr}\;\&\;\mathrm{drug}\;\mathrm{dev}\;\&\;\mathrm{buy}\;\mathrm{dr}\;1 \end{array}\right.$$

Equation (1) shows that outcome 14 (line 14) occurs if the disease is contracted, whereas outcome 5 or 6 (lines 5 or 6) occurs if the disease is not contracted, and person $$i$ buys 1 vaccine instead.

Inserting the parameter values into (7), vaccine company k ’s expected profit is

(2) $$\begin{array}{c} u_{1}=\\ \left\{ \begin{array}{c} \$0\;\mathrm{if}\;\mathrm{company}\;1\;\mathrm{does}\;\mathrm{not}\;\mathrm{develop}\;\mathrm{vaccine}\\ \begin{array}{c} -\$6.5\times 10^{7}\;\mathrm{if}\;\mathrm{company}\;1\;\mathrm{develops}\;\mathrm{vaccine}\;\mathrm{unsuccessfully}\left(g_{v1}=0\right)\\ \$3.35\times 10^{8}\;\mathrm{if}\;\mathrm{company}\;1\;\mathrm{develops}\;\mathrm{vaccine}\;\mathrm{successfully}\left(g_{v1}=1\right) \end{array} \end{array}\right.\\ u_{2}\\ =\left\{ \begin{array}{c} \$0\;\mathrm{if}\;\mathrm{company}\;2\;\mathrm{does}\;\mathrm{not}\;\mathrm{develop}\;\mathrm{vaccine}\\ \begin{array}{c} -\$6.5\times 10^{7}\;\mathrm{if}\;\mathrm{company}\;2\;\mathrm{develops}\;\mathrm{vaccine}\;\mathrm{unsuccessfully}\;\left(g_{v2}=0\right)\\ \$3.35\times 10^{8}\;\mathrm{if}\;\mathrm{company}\;2\;\mathrm{develops}\;\mathrm{vaccine}\;\mathrm{successfully}\;\left(g_{v2}=1\right) \end{array} \end{array}\right. \end{array}$$

and hence both vaccine companies develop vaccines.

Inserting the parameter values into (8), drug company $$j$’s expected profit is

(3) $$\begin{array}{c} U_{1}=\\ \left\{ \begin{array}{c} \$0\;\mathrm{if}\;\mathrm{company}\;1\;\mathrm{does}\;\mathrm{not}\;\mathrm{develop}\;\mathrm{drug}\\ \begin{array}{c} -\$1.25\times 10^{8}\;\mathrm{if}\;\mathrm{company}\;1\;\mathrm{develops}\;\mathrm{drug}\;\mathrm{unsuccessfully}\;\left(g_{d1}=0\right)\\ \$6.49\times 10^{8}\;\mathrm{if}\;\mathrm{company}\;1\;\mathrm{develops}\;\mathrm{drug}\;\mathrm{successfully}\;\left(g_{d1}=1\right) \end{array} \end{array}\right.\\ U_{2}=\\ \left\{ \begin{array}{c} \$0\;\mathrm{if}\;\mathrm{company}\;2\;\mathrm{does}\;\mathrm{not}\;\mathrm{develop}\;\mathrm{drug}\\ \begin{array}{c} -\$2.76\times 10^{9}\;\mathrm{if}\;\mathrm{company}\;2\;\mathrm{develops}\;\mathrm{drug}\;\mathrm{unsuccessfully}\;\left(g_{d2}=0\right)\\ \$1.42\times 10^{10}\;\mathrm{if}\;\mathrm{company}\;2\;\mathrm{develops}\;\mathrm{drug}\;\mathrm{successfully}\;\left(g_{d2}=1\right) \end{array} \end{array}\right. \end{array}$$

and hence both drug companies develop drugs.

The donor’s expected utility depends on a few additional parameters. We consider a country with $$N = 200$ million persons, where G=50 million persons choose safe behavior and L=70 million persons choose risky behavior while not contracting the disease. The indicator parameters in (10) are $I_{\mathrm{vk}}=I_{\mathrm{dj}}=1$ , j,k=1,2 , since both vaccine companies develop vaccines and both drug companies develop drugs. Inserting into (10), the donor’s expected utility depends on the sum of the N persons’ benefits $H_{i},E_{i},V_{\mathrm{ik}},D_{i},R_{i}$ spread across the 14 outcomes, accounting for nature’s probabilistic choices, and subtracting the donor’s cost of subsidy choices of $y_{k}$ , $s_{k}$ , $Y_{j}$ , $S_{j}$ , that is,

(4) $$V=\$\left(\begin{array}{c} 5.16700\times 10^{13}-0.13\times 10^{9}I_{v2}y_{2}-4.00\times 10^{8}s_{2}\\ -0.13\times 10^{9}I_{v1}y_{1}-4.00\times 10^{8}s_{1}\\ \begin{array}{c} -5.52\times 10^{9}I_{d2}Y_{2}-1.699\times 10^{10}S_{2}\\ -0.25\times 10^{9}I_{d1}Y_{1}-7.742\times 10^{8}S_{1} \end{array} \end{array}\right)=\$5.16577\times 10^{13}$$

where we have illustrated the role of the donor’s 8 strategic choice variables $${y_k} = {s_k} = {Y_j} = {S_j} = 0.5$ and the 4 indicator variables $I_{\mathrm{vk}}=I_{\mathrm{dj}}=1$ , j,k=1,2 .

#### Alternative scenarios with outcomes 1–4, 7–13

##### Person i

If person i does not contract the disease, instead of outcomes 5 and 6 in the benchmark scenario, outcome 4 occurs if only vaccine 1 is developed, for example, because vaccine company 2 does not succeed developing it. Outcome 3 occurs if only vaccine 2 is developed. Outcome 2 occurs if ${\left(1-q\left(\left(m_{1}\left(t\right)+m_{2}\left(t\right)\right)/N\right)\right)}^{\mathrm{rn}}E_{i}=\$729000$ million in (1) and (9) increases to exceed $W_{i}=\$799990$ for outcomes 5 and 6. That can happen if person i ’s utility $E_{i}=\$1$ million of risky behavior increases above $E_{i}=\$1.097380\times 10^{6}$ (which makes person i indifferent between outcomes 2 and 5 and 6) or the disease contraction probability $q\left(\left(m_{1}\left(t\right)+m_{2}\left(t\right)\right)/N\right)$ at time t in period 2 decreases. That, in turn, depends on the number $m_{k}\left(t\right)$ of persons having bought and been vaccinated by vaccine k , which depends on the other players’ decisions based on the prices of developing, producing, and selling vaccines. Outcome 2 can also occur if the vaccines become too expensive, that is, if $c_{k}$ increases sufficiently above $c_{k}=\$20$ , which depends on the other players, or person i ’s utility $V_{\mathrm{ik}}$ of vaccine k vaccination decreases sufficiently below $V_{\mathrm{ik}}=\$0.8$ million. For example, if the vaccine cost increases from $c_{k}=\$20$ to $c_{k}=\$142000$ , person i becomes indifferent between outcomes 2 and 5 and 6. Outcome 1 occurs if person i ’s utility $H_{i}=\$0.5$ million of safe behavior increases. In particular, if it increases to $H_{i}=\$799990$ , it equals or exceeds all the other outcomes 2–14, which may be possible for some persons, implying safe behavior.

If person i contracts the disease, instead of outcome 14 in the benchmark scenario, outcome 13 occurs if drug 2 (ivermectin) becomes cheaper than drug 1 (hydroxychloroquine), that is, $C_{2}<C_{1}$ . Outcome 11 with the same expected utility as outcome 14 occurs if only drug 1 is developed. Outcome 9 with the same expected utility as outcome 13 occurs if only drug 2 is developed. We then consider the outcomes in which player i does not buy drug j . Outcome 12 occurs if both drugs become too expensive when compared with the benefits, that is, $C_{1}$ and $C_{2}$ increase sufficiently above $C_{1}=\$38.71$ and $C_{2}=\$849.74$ , which depends on the other players. Outcome 12 may thus also occur if the benefit $\left(1-w_{j}\right)D_{i}+w_{j}R_{i}$ in outcomes 13 and 14 decreases. That benefit decreases when person i ’s utility $R_{i}=\$0.2$ million when recovering from disease decreases, or its disease recovery probability $w_{j}=0.95$ with drug j decreases, or its negative utility $D_{i}=-\$7$ million of death becomes more negative. Outcome 10 occurs when company 2 does not produce the drug but player 1 still finds drug 1 too expensive. Outcome 8 occurs when company 1 does not produce the drug but player 1 still finds drug 2 too expensive. Outcome 7 occurs when no company produces a drug, as discussed below, and then person i cannot buy it.

##### Vaccine company k _._

If vaccine company k does not develop the vaccine successfully, that is, $g_{\mathrm{vk}}=0$ , its expected profit $m_{k}c_{k}-{\left(m_{k}b_{k}\right)}^{a_{k}}-\left(1-y_{k}\right)f_{k}=\$3.35\times 10^{8}$ in row 3 in (2) for our benchmark scenario does not occur. That expected profit has a positive term $m_{k}c_{k}$ , which decreases if the cost $c_{k}=\$20$ of buying vaccine k decreases or the number $m_{k}=20$ million of persons buying vaccine k decreases (which depends on person i ’s decision). Vaccine company k ’s expected profit $u_{k}$ also has a cost term ${\left(m_{k}b_{k}\right)}^{a_{k}}+\left(1-y_{k}\right)f_{k}$ if $g_{\mathrm{vk}}=1$ , which increases if the vaccine k development cost $f_{k}=\$0.13$ billion increases or if the vaccine k production cost $m_{k}b_{k}=\$5\times 20\times 10^{6}$ destined for the $m_{k}=20$ million persons increases. If vaccine k is free, that is, $c_{k}=\$0$ , vaccine company k ’s expected profit decreases to $u_{k}=-\$6.501\times 10^{7}$ , causing vaccine k not to be produced. If the vaccine k development cost $f_{k}=\$0.13$ billion increases to $f_{k}=\$0.79998$ billion, vaccine company k ’s expected profit decreases to $u_{k}=\$0$ , causing it to be indifferent between producing and not producing the vaccine. If row 3 in (2) does not occur, outcomes 3, 4, 5, and 6 are impossible. Person i must then choose between safe behavior with utility $H_{i}=\$0.5$ million (outcome 1) or risky behavior with expected utility hoping for no disease contraction (outcome 2), or outcomes 7–14 with disease contraction.

##### Drug company

If drug company *j* does not develop the vaccine successfully, that is, *g*_*dj*_ = 0, its expected profit *M_j_C_j_−(M_j_B_j_)A_j_−(1−Y_j_) F_j_* in rows 2 and 3 in (8), that is, *U*_1_ = $6.49 × 10^8^ in row 3 and *U*_2_ = $1.42 × 10^10^ in row 6 in (3), for our benchmark scenario does not occur. That expected profit has a positive term *M_j_C_j_* that decreases if the costs *C*_1_ = $38.71 and *C*_2_ = $849.74 of buying drugs 1 and 2 decreases or the number *M*_*j*_ = 20 million persons buying *j* drug decreases (which depends on person *i*’s decision). Drug company *j*’s expected profit *U_j_* also has a cost term (*M_j_B_j_*)*A_j_*− (1 –*Y_j_*)*F_j_* if *g_dj_* = 1, which increases if the drug development costs *F*_1_ = $0.25 billion and *F*_2_ = $5.52 billion increase or if the drug *j* production costs *M*_1_*B*_1_ = $9.68 × 20 × 10^6^ and *M*_2_*B*_2_ = $212.44 × 20 × 10^6^ for the *M_j_* = 20 million persons increase. If drug 1 is free, that is, C_1_ = 0, drug company 1’s expected profit decreases to *U*_1_ = −$1.251014 × 10^7^, drug 1 will not be produced. If the drug 1 development cost *F*_1_ = $0.25 billion increases to *F*_1_ = $1.548372 billion, drug company 1’s expected profit decreases to *U*_1_ = $0, causing it to be indifferent between producing and not producing the drug. If rows 3 and 6 in (3) do not occur, so that no drugs are produced, outcomes 8–14 are impossible. Person *i* then has no choice causing outcome 7 with expected utility *W*_i_ = (1 −*x*)*D*_*i*_+*xR*_*i*_ = −$520,000. For person *i* to actively choose outcome 7, if it had a choice, its utility *R_i_* = $0.2 million when recovering from disease and its disease recovery probability *x* = 0.9 without a drug would have to be high, and its utility *D_i_* = −$7 million of death would have to be less negative.

##### The donor

If the donor does not choose its 8 subsidy fractions $Y_{j}=S_{j}=y_{k}=s_{k}=0.5$ , j,k=1,2 , that may affect all the other players’ decisions, potentially causing our benchmark scenario not to occur. That is, person i will incur higher costs $c_{k}$ and $C_{j}$ of buying vaccine k and drug j , potentially causing no vaccines and drugs to be bought. Vaccine company k will incur higher costs $f_{k}$ and $b_{k}$ of developing and producing vaccine k , potentially causing no vaccines to be produced. Drug company j will incur higher costs $F_{j}$ and $B_{j}$ of developing and producing drug j , potentially causing no drugs to be produced. For example, if the donor refrains from all subsidies, that is, $Y_{j}=S_{j}=y_{k}=s_{k}=0$ , the vaccine and drug companies are especially affected. Their expected profits in (2) and (3) decrease to

(5) $$\begin{array}{c} u_{1}=\\ \left\{ \begin{array}{c} \$0\;\mathrm{if}\;\mathrm{company}\;1\;\mathrm{does}\;\mathrm{not}\;\mathrm{develop}\;\mathrm{vaccine}\\ \begin{array}{c} -\$1.3\times 10^{8}\;\mathrm{if}\;\mathrm{company}\;1\;\mathrm{develops}\;\mathrm{vaccine}\;\mathrm{unsuccessfully}\;(g_{v1}=0)\\ \$2.70\times 10^{8}\;\mathrm{if}\;\mathrm{company}\;1\;\mathrm{develops}\;\mathrm{vaccine}\;\mathrm{successfully}\;(g_{v1}=1) \end{array} \end{array}\right.\\ u_{2}=\\ \left\{ \begin{array}{c} \$0\;\mathrm{if}\;\mathrm{company}\;2\;\mathrm{does}\;\mathrm{not}\;\mathrm{develop}\;\mathrm{vaccine}\\ \begin{array}{c} -\$1.3\times 10^{8}\;\mathrm{if}\;\mathrm{company}\;2\;\mathrm{develops}\;\mathrm{vaccine}\;\mathrm{unsuccessfully}\;(g_{v2}=0)\\ \$2.70\times 10^{8}\;\mathrm{if}\;\mathrm{company}\;2\;\mathrm{develops}\;\mathrm{vaccine}\;\mathrm{successfully}\;(g_{v2}=1) \end{array} \end{array}\right. \end{array}$$

(6) $$\begin{array}{c} U_{1}=\\ \left\{ \begin{array}{c} \$0\;\mathrm{if}\;\mathrm{company}\;1\;\mathrm{does}\;\mathrm{not}\;\mathrm{develop}\;\mathrm{drug}\\ \begin{array}{c} -\$2.5\times 10^{8}\;\mathrm{if}\;\mathrm{company}\;1\;\mathrm{develops}\;\mathrm{drug}\;\mathrm{unsuccessfully}\;\left(g_{d1}=0\right)\\ \$5.24\times 10^{8}\;\mathrm{if}\;\mathrm{company}\;1\;\mathrm{develops}\;\mathrm{drug}\;\mathrm{successfully}\;\left(g_{d1}=1\right) \end{array} \end{array}\right.\\ U_{2}=\\ \left\{ \begin{array}{c} \$0\;\mathrm{if}\;\mathrm{company}\;2\;\mathrm{does}\;\mathrm{not}\;\mathrm{develop}\;\mathrm{drug}\\ \begin{array}{c} -\$5.52\times 10^{9}\;\mathrm{if}\;\mathrm{company}\;2\;\mathrm{develops}\;\mathrm{drug}\;\mathrm{unsuccessfully}\;\left(g_{d2}=0\right)\\ \$1.15\times 10^{10}\;\mathrm{if}\;\mathrm{company}\;2\;\mathrm{develops}\;\mathrm{drug}\;\mathrm{successfully}\;\left(g_{d2}=1\right) \end{array} \end{array}\right. \end{array}$$

With this change, the vaccines and drugs are still produced, but the companies’ profit margins are lower and may become negative if other parameter values change adversely.

## Discussion, Limitations, Future Research, and Literature Review

### Discussion

The model illustrates the strategic interaction between $$N$ persons choosing risky or safe behavior, 2 vaccine companies choosing whether or not to produce vaccines, 2 drug companies choosing whether or not to produce drugs, and a donor. Further influence is made by nature. This strategic interaction has 14 outcomes, illustrated in (9), (1), Table 1 (Supplementary Appendix D), among others. The optimal strategic choices for each player, exemplified in the previous section, constitute useful information for each type of player in the game for selecting an appropriate strategy to address the pandemic.

The parameter estimation in the section “Numerical example estimating the parameters” in the Results section illustrates how each of these 14 outcomes can be realized. The article shows how each person chooses risky or safe behavior dependent on the expected benefits and costs of the various outcomes and nature’s choice of the probability of disease contraction. That probability is of particular interest related to the finding of Galárraga et al.^7^ of 7000 HIV infections per day in their study.

We illustrate a benchmark scenario in which vaccines and drugs are produced and person i buys vaccine 1 or vaccine 2 (outcomes 5 or 6) if the disease is not contracted and buys drug 1 if the disease is contracted (outcome 14). Person i ’s choice of whether or not to buy a vaccine or a drug depends on weighing the benefits against the costs. Alternative scenarios are presented in which person i either buys no vaccine or no drug (outcomes 1–4, 7–13), which is more likely for risk-seeking persons. That happens, to the extent quantified in the examples in the previous section, if the probability of disease contraction is low (a risk-seeking person may consider the probability as negligible), if the vaccine or drug becomes too expensive, affected by whether the donor subsidizes. It also happens if the utility of vaccination decreases (e.g., because the virus mutates or vaccination has side effects) or if the expected utilities of risky behavior, recovery, and death are too low with the drug compared with not applying the drug. Person i may also not buy drug j if it does not sufficiently increase nature’s probability of recovery, which is $w_{j}$ with drug j and x without a drug, $x\leq w_{j}$ . To make these decisions optimally, each person must be appropriately informed, for example, as recommended by Hogan et al.^15^

The parameters and the players’ strategies change with changing markets, prices, diseases, persons’ preferences, demography, modes of interaction, technology, and so forth. In the benchmark scenario, the 4 companies produce vaccines and drugs. The companies’ choices also depend on weighing the benefits against the costs, which can be decreased as recommended by Granich et al.,^25^, Bärnighausen et al.,^26^ Forsythe et al.,^27^ and others, as discussed in the section “Treatment” in the Introduction. In the alternative scenarios, as quantified in the examples, vaccines and drugs are not developed and produced if the costs are too high, affected by whether the donor subsidizes, if the probability of successful development is too low, and if too few persons buy the vaccines or drugs at too low costs. As the persons’ and companies’ strategies change, the previous section also shows how the donor’s weighing of the benefits to the N persons versus the subsidization costs change. The donor’s adjustment of its subsidization of development, production, and purchases of vaccines and drugs addresses how Fitzpatrick et al.’s^6^ finding of underinvestment in public health can be remedied.

Societal changes in preferences, beliefs, demography, and modes of interaction may change the persons’ expected utilities of the various outcomes. More effective and cheaper drugs, produced and distributed more effectively, may affect the outcomes. Such factors affect which of the 14 outcomes occur. Understanding the impact of changes may enable players to choose better strategies and enable policy makers and others, not modeled in this article, to make good decisions.

The prices of vaccines and drugs are affected by many factors. Large volumes generally cause price reduction.^49^ COVID-19 is too recent to experience established price discovery for vaccines and drugs. Some drug manufacturers of antiretroviral drugs for HIV/AIDS, such as Merck, GlaxoSmithKline, and Bristol Myers Squibb, apply price tiers depending on the countries’ socioeconomic status. Some apply the World Bank definition of low, lower-middle, upper-middle, and high-income countries.^50^ Other companies apply their own classification.^51^ Prices are also influenced by procurement processes including third-party negotiations. For example, the Clinton Health Access Initiative^52^ negotiates procurement prices on behalf of its member countries with mainly generic manufacturers.

The article assumes that person i binarily chooses risky or safe behavior. In practice, that can be interpreted so that if person i ’s behavior is risky above a certain level, then the behavior is risky, and if it is risky below that level, then it is safe. Embedded in person i ’s choice is the environment in which person i makes its choice. For example, if person i chooses safe behavior when the environment is not accounted for and lives in a household where many other persons choose risky behavior, then a level may be exceeded so that person i ’s behavior is actually risky. Averaged over N persons, their binary choices between risky and safe behavior constitute an approximation, in which where a fraction p chooses risky behavior above the specified level and the remaining fraction 1−p chooses safe behavior. The approximation enables the simplification in Figure 2, in which only 2 arrows flow from person i ’s decision node in period 1. That simplification allows the analytical tractability in the subsequent equations, causing insights that may not be possible with a more complicated conception of how person i ’s risk attitude affects its behavior. One example of a generalization is to assume a probability distribution for how the N person’s behavior ranges from extremely safe to extremely risky. A continuum of arrows will then flow out from person i ’s decision node in period 1 in Figure 2, and the equations will have to be revised to account for the specified probability distribution.

No vaccine exists against HIV/AIDS, which emerged in 1983–1984, because of HIV’s high strain diversity. The HIV variability within one individual exceeds the worldwide variability in the influenza virus during one season. HIV’s high virus replication prevents recognition by antibodies.^53^ Five unsuccessful phase 3 vaccine efficacy trials have been performed against HIV, each costing more than US$100 million. Game theoretically, this means that the vaccine k development cost $f_{k}$ is prohibitively high and has hitherto been unsuccessful. Hence, the negative term $-\left(1-y_{k}\right)f_{k}$ in row 2 in (7) for vaccine company k gets a high absolute value, causing no vaccine production and thus outcome 2 in Figure 3 and Table 1 (Supplementary Appendix D), instead of outcomes 5 or 6 for COVID-19. Even if an HIV/AIDS vaccine were available, person i may not buy it if it assesses the disease contraction probability q to be low. Neither HIV/AIDS nor COVID-19 resolve in most cases, but HIV infection is less transmittable, whereas COVID-19 is very transmittable. If the disease contraction probability q decreases from q=0.1 in the benchmark scenario in the section “Benchmark scenario with outcomes 5,6,14” in the Results section to below q=0.07169 , person i gets a higher expected utility above $W_{i}=\$799990$ in row 2 in (1) and would thus choose outcome 2 even if a vaccine were available. For drugs, the situation is opposite. Drugs for HIV/AIDS have emerged since 1983–1984 at higher effectiveness and lower prices. Hence, the negative term $-\left(1-Y_{j}\right)F_{j}$ in row 2 in (8) for drug company j and the negative term $-\left(1-S_{j}\right)C_{j}$ in rows 13 and 14 in (9) for person i get low absolute values, more easily causing the outcomes 13 and 14 in Figure 4, in which company j produces the drug and person i buys it. For COVID-19, few drugs have emerged beyond hydroxychloroquine and ivermectin. The long-term success rate is not yet well understood.

### Limitations

Limitations of the article are related to different market conditions, technology, and preferences of the players over time. Vaccine and drug companies develop increased competence over time, while diseases change due to virus mutation. How donors sponsor and how nature chooses disease contraction, recovery, death, and whether vaccines and drugs are developed successfully also have impact. Some suggestions are made below for how future research may address such limitations.

### Future Research

The article assumes rational agents and conventional expected utility theory. Future research may verify the results assuming boundedly rational agents^54^ and alternatives, such as prospect theory, in which the expected utility is concave for gains, convex for losses, assigns excessive weight to low probability events, and insufficient weight to low-probability events.^55^

Future research may assess how a country’s economy, productivity, economic growth, and societal indicators such as income and health are affected by the factors analyzed in this article. Research may distinguish between persons according to age, sex, occupation, ethnicity, race, and so forth and model probability distributions for types of persons according to utilities for risky behavior, safe behavior, recovery from disease, and death.

Research may model different kinds of competition between multiple vaccine and drug companies; competition between multiple donors as strategic players; regulation by multiple regulatory agencies; model more players such as doctors, hospitals, regulators, and politicians; and account for more choices by nature, which may potentially be endogenized. For example, the disease contraction probability for infectious diseases may depend on how many persons have previously contracted the disease in each person’s various networks of family, work, leisure activities, and so on. The disease recovery probability with and without various vaccines and drugs may be endogenized by modeling the biological virus evolution processes.^21,56^

Research may model how people choose different kinds and degrees of risky and safe behavior before, during, and after various vaccines and drugs are produced and available. Such behaviors can be expected to depend on persons’ perceptions of the qualities and prices of vaccines and drugs and how vaccines and drugs are adjusted to the changing characteristics of various diseases. Future research should collect empirical data on a variety of infectious diseases, assess empirical support of the model in this article and other models, and develop further models.

## Conclusion

Three linked games for an infectious disease such as COVID-19 or HIV/AIDS are developed between N persons, 2 vaccine companies, 2 drug companies, and 1 donor as strategic players, impacted by nature. Fourteen outcomes determine each person’s expected utility, that is, for safe behavior (outcome 1), risky behavior without disease contraction and without vaccination (outcome 2), outcomes 3–6 if each of the 2 vaccines is produced and bought, and outcomes 7–14 if each of the 2 drugs is produced and bought or not bought. Applying backward induction, the game is solved accounting for the 14 outcomes and whether vaccines and drugs are produced and bought. The parameters are estimated based on early COVID-19 data for the BioNTech/Pfizer Comirnaty vaccine and the Moderna vaccine and the experimental drugs hydroxychloroquine and ivermectin. We illustrate how a person buys 1 of the vaccines if not contracting the disease and 1 of the drugs otherwise. We show how a person may not buy a drug or a vaccine if it is too expensive, how vaccine and drug companies may not produce if expected profits are low, and the donor’s impact.

The article illustrates how the players (i.e., persons, vaccine and drug companies, the donor, and nature) strike balances in a game-theoretic cost-benefit analysis that impacts which of the 14 outcomes arise. Illustrating how such balances are struck may improve society’s ability to handle infectious diseases and has managerial implications for running vaccine and drug companies, determining how a donor should subsidize, and how persons should manage their own health. More specifically, each player has a benefit and a cost. Each person incurs a cost of buying a vaccine or drug, which may be sponsored by a donor, weighted against probabilistic utilities associated with risky or safe behavior, vaccination, recovery, and death, affecting the person’s strategy. Relative to a benchmark scenario in which a person buys a vaccine or drug, we show how changing conditions may affect the person not to buy a vaccine or drug.

Each vaccine and drug company incurs a cost of developing and producing a vaccine or drug, successfully or unsuccessfully, potentially sponsored by a donor. The production cost depends on how many persons buy the vaccine or drug, which illustrates the linkage between the players. Each vaccine and drug company’s benefit is the price of the vaccine or drug, which also depends on how many persons buy it. We show how changing conditions may induce vaccine and drug companies not to produce vaccines and drugs. The donor’s expected utility equals the sum of the persons’ benefits across the 14 outcomes minus the cost of subsidizing the development and purchase of vaccines and drugs. The donor subsidizes based on weighing the benefit against the cost.

As a pandemic evolves, nature’s probabilities of disease contraction and recovery with and without a drug changes. This affects each person’s strategies of safe versus risky behavior and whether to buy a vaccine or drug. Each vaccine and drug company faces uncertainties as to whether a vaccine or drug can be successfully developed and produced, at what cost, how many persons will buy it, at which price, and how the sponsor may subsidize. The strong linkages between the players affect their strategies. Various scenarios are presented for how the players choose strategies associated with the 14 outcomes. Policy makers may assess the players’ strategies when designing broader societal strategies, potentially inducing players to choose the preferable outcomes among the 14 outcomes.

Future research may generalize the model to include various strategies for doctors, hospitals, advisors, insurance companies, and so on that also play a role in the health and political system. The persons’ strategies may be generalized to a continuum from extremely risky to extremely safe. More than 2 vaccine companies and 2 drug companies may be considered, with various forms of competition (e.g., on price and quality) between them.

## Supplemental Material

sj-docx-1-mdm-10.1177_0272989X211053563 – Supplemental material for A Game Theoretic Analysis of Competition Between Vaccine and Drug Companies during Disease Contraction and Recovery Click here for additional data file. Supplemental material, sj-docx-1-mdm-10.1177_0272989X211053563 for A Game Theoretic Analysis of Competition Between Vaccine and Drug Companies during Disease Contraction and Recovery by Kjell Hausken and Mthuli Ncube in Medical Decision Making

sj-docx-2-mdm-10.1177_0272989X211053563 – Supplemental material for A Game Theoretic Analysis of Competition Between Vaccine and Drug Companies during Disease Contraction and Recovery Click here for additional data file. Supplemental material, sj-docx-2-mdm-10.1177_0272989X211053563 for A Game Theoretic Analysis of Competition Between Vaccine and Drug Companies during Disease Contraction and Recovery by Kjell Hausken and Mthuli Ncube in Medical Decision Making

sj-docx-3-mdm-10.1177_0272989X211053563 – Supplemental material for A Game Theoretic Analysis of Competition Between Vaccine and Drug Companies during Disease Contraction and Recovery Click here for additional data file. Supplemental material, sj-docx-3-mdm-10.1177_0272989X211053563 for A Game Theoretic Analysis of Competition Between Vaccine and Drug Companies during Disease Contraction and Recovery by Kjell Hausken and Mthuli Ncube in Medical Decision Making

sj-docx-4-mdm-10.1177_0272989X211053563 – Supplemental material for A Game Theoretic Analysis of Competition Between Vaccine and Drug Companies during Disease Contraction and Recovery Click here for additional data file. Supplemental material, sj-docx-4-mdm-10.1177_0272989X211053563 for A Game Theoretic Analysis of Competition Between Vaccine and Drug Companies during Disease Contraction and Recovery by Kjell Hausken and Mthuli Ncube in Medical Decision Making

## Notes

i The cost-effectiveness of an intervention to prevent HIV infection is further analyzed in references 8–13. These authors contended that decreasing HIV transmission should involve combination prevention through a mix of communication channels, and that HIV prevention procedures should be agreed upon, funded, implemented, measured, and achieved. The HIV Modelling Consortium Treatment as Prevention Editorial Writing Group^14^ recommends cooperation between disciplines such as epidemiology, economics, demography, statistics, and biology, supported by mathematical modeling, to facilitate evidence-based HIV prevention decision making to ensure the optimal use of antiretroviral therapy.

ii Alistar and Brandeau^16^ analyzed characteristics in a variety of models, enabling parameter customization and accounting for uncertainty, to scale up decision making about HIV prevention and treatment. Boily et al.^17^ found that decreasing the HIV incidence over 2 to 3 y of cluster randomized controlled treatment as prevention (TasP) programs is challenging unless interventions are scaled up to reach key populations. They recommend mathematical modeling to conduct interim analyses. Hecht et al.^18^ and Izazola-Licea et al.^19^ assessed the financing of the response to HIV/AIDS in low-income and middle-income countries. Goldie et al.^20^ recommended trimethoprim–sulfamethoxazole prophylaxis and antiretroviral therapy, applying clinical criteria alone or in combination with CD4 testing, to improve the cost-effectiveness of HIV treatment in resource-poor communities. Moxnes and Hausken^21^ applied differential time equations to acute virus influenza A infections affecting the immune system, illustrated with the 1918 Spanish flu virus H1N1. For further studies, see references 22–24.

iii As categorized by Cornish,^38^ a donor may be 1 or several governments, nongovernmental organizations, civil society organizations, philanthropic organizations, private sector donors, or other donors, acting bilaterally or multilaterally. We assume that the donors resolve their collective action problem.

iv On January 4, 2021, BioNTech/Pfizer announced the discounted price of $10 per dose for the Comirnaty vaccine for users in South Africa.^40^

v The common method is to assess how persons strike balances between health risks and rewards, for example, weighing wages against death risk in the labor market.
