# Supplementary material for: A Game Theoretic Analysis of Competition Between Vaccine and Drug Companies during Disease Contraction and Recovery
Source: Med Decis Making. 2021 Nov 5;42(5):571–86. doi: 10.1177/0272989X211053563 (PMC9189729; doi:10.1177/0272989X211053563)
Supplement: sj-docx-1-mdm-10.1177_0272989X211053563 – Supplemental material for A Game Theoretic Analysis of Competition Between Vaccine and Drug Companies during Disease Contraction and Recovery [file sj-docx-1-mdm-10.1177_0272989X211053563.docx]

Appendix A Nomenclature

Parameters

$N$ Number of persons

$G$ Number of persons choosing safe behavior

$L$ Number of persons choosing risky behavior while not contracting the disease

$m_{k}\left( t \right)$ Number of persons having bought and been vaccinated by vaccine $k$, $k=1,2$, at time $t$.

$m_{k}=\lim_{t\to\infty} m_{k}\left( t \right)$ Number of persons buying vaccine $k$, $k=1,2$

$c_{k}$ Vaccine $k$ purchasing cost for person $i$, $i=1,\ldots,N$, $k=1,2$

$b_{k}$ Vaccine $k$ production cost for vaccine company $k$ destined for person $i$, $i=1,\ldots,N$, $k=1,2$

$a_{k}$ Exponential parameter scaling vaccine $k$ production cost, $a_{k}\geq0$, $k=1,2$

$f_{k}$ Vaccine $k$ development cost, $k=1,2$

$I_{vk}$ Indicator parameter for whether vaccine $k$ is produced ($I_{vk}=1$) or not ($I_{vk}=0$), $k=1,2$

$n$ Number of returns from period 2 in Figure 3 to period 1 in Figure 2 when assessing $E_{i}$, $n\geq0$

$r$ Scaling parameter for $n$, $r\geq0$

$M_{j}$ Number of persons buying drug $j$, $j=1,2$

$C_{j}$ Drug $j$ purchasing cost for person $i$, $i=1,\ldots,N$, $j=1,2$

$B_{j}$ Drug $j$ production cost for drug company $j$ destined for person $i$, $i=1,\ldots,N$, $j=1,2$

$A_{j}$ Exponential parameter scaling drug $j$ production cost, $A_{j}\geq0$, $j=1,2$

$F_{j}$ Drug $j$ development cost, $j=1,2$

$I_{dj}$ Indicator parameter for whether drug $j$ is produced ($I_{dj}=1$) or not ($I_{dj}=0$), $j=1,2$

$E_{i}$ Person $i$’s utility of risky behavior, $i=1,\ldots,N$

$V_{ik}$ Person $i$’s utility of vaccine $k$ vaccination, $V_{ik}<E_{i}$, $i=1,\ldots,N$, $k=1,2$

$H_{i}$ Person $i$’s utility of safe behavior, $H_{i}<V_{ik}$, $i=1,\ldots,N$

$R_{i}$ Person $i$’s utility when recovering from disease, ${0<R}_{i}<H_{i}$, $i=1,\ldots,N$

$D_{i}$ Person $i$’s utility of death, ${0>D}_{i}<R_{i}>0$, $i=1,\ldots,N$

Independent variable

$t$ Time, $t\geq0$

Strategic choices by person $i$, $i=1,\ldots,N$

Choice between risky and safe behavior in period 1 in Figure 2

Choice whether to buy or not buy drug $j$ in period 2 in Figure 4, $j=1,2$

Choice whether to buy or not buy vaccine $k$ in period 2 in Figure 3, $k=1,2$

Strategic choice by vaccine company $k$, $k=1,2$

Choice whether to produce vaccine $k$ in period 1 in Figure 3, $k=1,2$

Strategic choice by drug company $j$, $j=1,2$

Choice whether to produce drug $j$ in period 1 in Figure 4, $j=1,2$

Strategic choices by donor

$y_{k}$ Subsidy fraction of vaccine $k$ development cost $f_{k}$ in period 1 in Figure 3, ${0\leq y}_{k}\leq1$, $k=1,2$

$s_{k}$ Subsidy fraction of vaccine $k$ purchasing cost $c_{k}$ for person $i$ in period 2 in Figure 3, ${0\leq s}_{k}\leq1$, $k=1,2$

$Y_{j}$ Subsidy fraction of drug $j$ development cost $F_{j}$ in period 1 in Figure 3, ${0\leq Y}_{j}\leq1$, $j=1,2$

$S_{j}$ Subsidy fraction of drug $j$ purchasing cost $C_{j}$ for person $i$ in period 2 in Figure 4, ${0\leq S}_{j}\leq1$, $j=1,2$

Strategic choices by Nature

$q\left( \left( m_{1}\left( t \right)+m_{2}\left( t \right) \right)/N \right)$ Disease contraction probability at time $t$ in period 2 in Figure 2, $0\leq q\leq1$

$x$ Disease recovery probability without drug in period 3 in Figure 4, $0\leq x\leq1$

$w_{j}$ Disease recovery probability with drug $j$ in period 3 in Figure 4, $0\leq x\leq w_{j}\leq1$, $j=1,2$

$g_{vk}$ Probability that vaccine $k$ is developed successfully, $0\leq g_{vk}\leq1$, $k=1,2$

$g_{dj}$ Probability that drug $j$ is developed successfully, $0\leq g_{dj}\leq1$, $j=1,2$

Dependent variables

$p$ Fraction of the $N$ persons choosing risky behavior, $0\leq p\leq1$

$W_{i}$ Person $i$’s expected utility, $i=1,\ldots,N$

$U_{j}$ Drug company $j$’s expected profit, $j=1,2$

$u_{k}$ Vaccine company $k$’s expected profit, $k=1,2$

$u_{D}$ Donor’s expected utility
