# Supplementary material for: A Game Theoretic Analysis of Competition Between Vaccine and Drug Companies during Disease Contraction and Recovery
Source: Med Decis Making. 2021 Nov 5;42(5):571–86. doi: 10.1177/0272989X211053563 (PMC9189729; doi:10.1177/0272989X211053563)
Supplement: sj-docx-2-mdm-10.1177_0272989X211053563 – Supplemental material for A Game Theoretic Analysis of Competition Between Vaccine and Drug Companies during Disease Contraction and Recovery [file sj-docx-2-mdm-10.1177_0272989X211053563.docx]

Appendix B More extensive game description

### *B1 Vaccine company* $k$*’s behavior, benefits, and costs*

Vaccine company $k$, $k=1,2$, has one strategic choice variable. It chooses in period 1 in Figure 3 either to develop the vaccine at cost $f_{k}$ subsidized by a donor with a fraction $y_{k}$, ${0\leq y}_{k}\leq1$, or not to develop the vaccine at no cost. Thus vaccine company $k$ pays $\left( 1-y_{k} \right)f_{k}$ for vaccine development. If vaccine $k$ is produced, person $i$ chooses in period 2 in Figure 3 either to buy it at cost $c_{k}$ subsidized by the donor (or multiple donors interpreted as one collective unit) with a fraction $s_{k}$, ${0\leq s}_{k}\leq1$, after which the game ends, or not to buy it. If person $i$ does not buy vaccine $k$, or vaccine $k$ is not produced, the game returns to period 1 in Figure 2.

Vaccine company $k$’s expected profit, $k=1,2$, depends on how many persons $m_{k}$ buy vaccine $k$, the vaccine price $c_{k}$, the vaccine $k$ development cost $f_{k}$, the donor’s subsidy fraction $y_{k}$ of the vaccine $k$ development cost $f_{k}$, and whether vaccine $k$ is developed successfully expressed with Nature’s probability $g_{vk}$. We assume that $m_{k}$ persons buy vaccine $k$, causing one of the outcomes 3,4,5,6. Vaccine company $k$ earns zero profit if the vaccine is not developed, so that stage 1 in Figure 1 is not entered. Vaccine company $k$ earns negative profit $-\left( 1-Y_{j} \right)F_{j}$ if it incurs development costs, subsidized by a donor with the fraction $Y_{j}$, but fails to develop the vaccine successfully, so that stage 2 in Figure 1 is not entered. Vaccine company $k$’s expected profit is

| $u_{k}=\left\{ \begin{matrix} 0 if company k does not develop the vaccine \\ \begin{matrix} g_{vk}\left[ m_{k}c_{k}-\left( m_{k}b_{k} \right)^{a_{k}} \right]-\left( 1-y_{k} \right)f_{k} if company k develops \\ the vaccine successfully with probability g_{vk} \end{matrix} \end{matrix} \right.$ | (7) |
| --- | --- |

where $b_{k}$ is the vaccine production cost for vaccine company $k$ in the amount required for person $i$, $i=1,\ldots,N$, and $a_{k}$, $a_{k}\geq0$, scales the production cost. In (7), the positive term ${g_{vk}m}_{k}c_{k}$ expresses vaccine company $k$’s expected profit from selling the vaccine at the price $c_{k}$ to $m_{k}$ buyers when the probability is $g_{vk}$ that the vaccine is developed successfully. The negative term $g_{vk}\left( m_{k}b_{k} \right)^{a_{k}}$ is vaccine company $k$’s expected cost of producing the vaccine for $m_{k}$ buyers when the probability is $g_{vk}$ that the vaccine is developed successfully. When vaccine $k$ is not developed successfully, i.e. $g_{vk}=0$, it cannot be produced, and then $g_{vk}\left( m_{k}b_{k} \right)^{a_{k}}=0$. The negative term $\left( 1-y_{k} \right)f_{k}$ is vaccine company $k$’s vaccine development cost adjusted by the donor subsidy fraction $y_{k}$. That term is not multiplied with $g_{vk}$ since the cost is incurred regardless of whether the vaccine development is successful or not. The production cost is linear when $a_{k}=1$, concave when $0<a_{k}<1$, and convex when $a_{k}>1$.

### *B2 Drug company* $j$*’s behavior, benefits, and costs*

Drug company $j$, $j=1,2$, has one strategic choice variable. It chooses in period 1 in Figure 4 either to develop the drug at cost $F_{j}$ subsidized by a donor with a fraction $Y_{j}$, ${0\leq Y}_{j}\leq1$, or not to develop the drug at no cost. Thus drug company $j$ pays $\left( 1-Y_{j} \right)F_{j}$ for drug development. Drug design, development and approval can take 10-15 years, but usually less (Lansdowne, 2020). If no drug company produces drugs, or person $i$ does not purchase a drug in period 2 in Figure 4, Nature chooses in period 3 in Figure 4 recovery with probability $x$ causing positive utility $R_{i}$, ${0<R}_{i}<H_{i}$, or death (or substantially decreased life quality) with probability $1-x$ causing negative utility $D_{i}$, $0>D_{i}<R_{i}>0$, after which the game ends. If drug $j$ is produced, person $i$ chooses either to buy it at cost $C_{j}$ subsidized by the donor (or multiple donors interpreted as one collective unit) with a fraction $S_{j}$, ${0\leq S}_{j}\leq1$, or not to buy it. If person $i$ buys drug $j$, paying $\left( 1-S_{j} \right)C_{j}$, Nature chooses recovery or death with probabilities $w_{j}$ and ${1-w}_{j}$ respectively. If person $i$ does not buy drug $j$, Nature chooses recovery or death with probabilities $x$ and $1-x$ respectively. The probabilities $p,q,x,w_{j}$ are independent.

Drug company $j$’s expected profit, $j=1,2$, depends on how many persons $M_{j}$ buy drug $j$, the drug price $C_{j}$, the drug $j$ development cost $F_{j}$, the donor’s subsidy fraction $Y_{j}$ of the drug $j$ development cost $F_{j}$, and whether drug $j$ is developed successfully expressed with Nature’s probability $g_{dj}$. We assume that $M_{j}$ persons buy drug $j$, causing one of the outcomes 9,11,13,14. Drug company $j$ earns zero expected profit if the drug is not developed, so that stage 1 in Figure 1 is not entered. Drug company $j$ earns negative expected profit $-\left( 1-Y_{j} \right)F_{j}$ if it incurs development costs, subsidized by a donor with the fraction $Y_{j}$, but fails to develop the drug successfully, so that stage 2 in Figure 1 is not entered. Drug company $j$’s expected profit is

| $U_{j}=\left\{ \begin{matrix} 0 if company j does not develop the drug \\ \begin{matrix} g_{dj}\left[ M_{j}C_{j}-\left( M_{j}B_{j} \right)^{A_{j}} \right]-\left( 1-Y_{j} \right)F_{j} if company j develops \\ the drug successfully with probability g_{dj} \end{matrix} \end{matrix} \right.$ | (8) |
| --- | --- |

where $B_{j}$ is the drug production cost for drug company $j$ in the amount required for person $i$, $i=1,\ldots,N$, and $A_{j}$, $A_{j}\geq0$, scales the production cost. In (8), the positive term ${g_{dj}M}_{j}C_{j}$ expresses drug company $j$’s expected profit from selling the drug at the price $C_{j}$ to $M_{j}$ buyers when the probability is $g_{dj}$ that the drug is developed successfully. The negative term ${g_{dj}\left( M_{j}B_{j} \right)}^{A_{j}}$ is drug company $j$’s expected cost of producing the drug for $M_{j}$ buyers when the probability is $g_{dj}$ that the drug is developed successfully. The negative term $\left( 1-Y_{j} \right)F_{j}$ is drug company $j$’s drug development cost adjusted by the donor subsidy fraction $Y_{j}$. That term is not multiplied with $g_{dj}$ since the cost is incurred regardless of whether the drug development is successful or not. The production cost is linear when $A_{j}=1$, concave (economy of scale) when $0<A_{j}<1$, and convex (diseconomy of scale) when $A_{j}>1$.

B3 Person $i$, $i=1,\ldots,N$

If person $i$, *i=1,…,N*, has not contracted the disease, it chooses in period 2 in Figure 3 whether or not to buy vaccine $k$, $k=1,2$, if it is available, potentially subsidized by a donor, and buys maximally one vaccine. If person $i$ does not buy vaccine $k$, or vaccine $k$ is not produced, the game returns to period 1 in Figure 2 where person $i$ either chooses safe behavior, or the disease is not contracted with probability $1-q\left( \left( m_{1}\left( t \right)+m_{2}\left( t \right) \right)/N \right)$. Defining $n$ as the number of returns to period 1 in Figure 2, $\left( 1-q\left( \left( m_{1}\left( t \right)+m_{2}\left( t \right) \right)/N \right) \right)^{rn}E_{i}$ expresses person $i$’s expected utility if not contracting the disease after $n$ returns to period 1 in Figure 2, where $r$, $r\geq0$, is a scaling parameter. That is, person $i$ receives utility $E_{i}$ when arriving in period 2 in Figure 3 the first time and not buying drugs, i.e. after $n=0$ returns to period 1 in Figure 2; receives expected utility $\left( 1-q\left( \left( m_{1}\left( t \right)+m_{2}\left( t \right) \right)/N \right) \right)^{n}E_{i}$ when arriving in period 2 in Figure 3 the second time without buying drugs, i.e. after $n=1$ returns to period 1 in Figure 2; receives expected utility $\left( 1-q\left( \left( m_{1}\left( t \right)+m_{2}\left( t \right) \right)/N \right) \right)^{2n}E_{i}$ when arriving in period 2 in Figure 3 the third time without buying drugs, i.e. after $n=2$ returns to period 1 in Figure 2 etc. The time between each return to period 1 in Figure 2 when not buying the vaccine depends on the nature of the disease. For a disease such as Covid-19 we may envision the time between each return to period 1 in Figure 2 to be in the magnitude of months. The more times person $i$ returns to period 1 in Figure 2, causing $n$ to increase, and thus causing more time to elapse without being vaccinated, the lower becomes person $i$’s expected utility $\left( 1-q\left( \left( m_{1}\left( t \right)+m_{2}\left( t \right) \right)/N \right) \right)^{rn}E_{i}$, which may eventually become lower than the utility $V_{ik}$ of being vaccinated with vaccine $k, k=1,2$.

If person $i$, *i=1,…,N*, has contracted the disease, it chooses in period 2 in Figure 4 whether or not to buy drug $j$, $j=1,2$, if it is available, potentially subsidized by a donor, and buys maximally one drug.

Figure 2 shows person $i$’s 14 outcomes. Outcome 1 occurs if person i chooses safe behavior. Outcomes 2-6 occur if person $i$ chooses risky behavior without disease contraction causing transition to Figure 3. Outcomes 7-14 occur if person $i$ chooses risky behavior with disease contraction causing transition to Figure 4, where Nature’s probabilistic choice of recovery or death is not counted. Person $i$’s expected utility for these 14 outcomes is

| $W_{i}=\left\{ \begin{matrix} \begin{matrix} \begin{matrix} \begin{matrix} \begin{matrix} H_{i} if safe behavior \& no disease contraction \& no vaccination \\ \begin{matrix} \left( 1-q\left( \left( m_{1}\left( t \right)+m_{2}\left( t \right) \right)/N \right) \right)^{rn}E_{i} if rb \& no dis contr \& no vaccination \\ V_{i2}-\left( 1-s_{2} \right)c_{2} if rb \& vaccine 2 development \& vaccination \\ \begin{matrix} V_{i1}-\left( 1-s_{1} \right)c_{1} if rb \& vaccine 1 development \& vaccination \\ V_{i2}-\left( 1-s_{2} \right)c_{2} if rb \& vaccines 1\&2 develop \& vaccine 2 vaccination \\ V_{i1}-\left( 1-s_{1} \right)c_{1} if rb \& vaccines 1\&2 develop \& vaccine 1 vaccination \end{matrix} \end{matrix} \end{matrix} \\ \left( 1-x \right)D_{i}+xR_{i} if risky beh \& disease contr \& no drug development \\ \left( 1-x \right)D_{i}+xR_{i} if risky beh \& dis contr \& drug dev \& not buy drug 2 \end{matrix} \\ \left( 1-w_{2} \right)D_{i}+w_{2}R_{i}-\left( 1-S_{2} \right)C_{2} if rb \& dis contr \& dr dev \& buy drug 2 \\ \left( 1-x \right)D_{i}+xR_{i} if rb \& dis contr \& drug dev \& not buy drug 1 \end{matrix} \\ \left( 1-w_{1} \right)D_{i}+w_{1}R_{i}-\left( 1-S_{1} \right)C_{1} if rb \& dis contr \& drug dev \& buy dr 1 \\ \left( 1-x \right)D_{i}+xR_{i} if rb \& dis contr \& drug dev \& not buy drugs 1 or 2 \end{matrix} \\ \left( 1-w_{2} \right)D_{i}+w_{2}R_{i}-\left( 1-S_{2} \right)C_{2} if rb \& dis contr \& drug dev \& buy dr 2 \\ \left( 1-w_{1} \right)D_{i}+w_{1}R_{i}-\left( 1-S_{1} \right)C_{1} if rb \& dis contr \& drug dev \& buy dr 1 \end{matrix} \right.$ | (9) |
| --- | --- |

where “rb” means “risky behavior” and the other text abbreviations are self-explanatory. Person $i$’s expected utility is in $ since its vaccine $k$ purchasing cost $c_{k}$, drug $j$ purchasing cost $C_{j}$, and utilities $E_{i}$,$V_{ik}$,$H_{i}$,$R_{i}$,$D_{i}$ are denominated in $. Line 1 in (9) applies to a risk averse person $i$ attributing high utility $H_{i}$ to safe behavior and low expected utility to the outcomes of risky behavior, when accounting for the probabilities $x$ and $w_{j}$, $j=1,2$, drug production by the two companies, and costs. Line 2 applies when Nature chooses no disease contraction, and person $i$ does not get vaccinated, causing utility $H_{i}$. Lines 3 and 5, causing utility $V_{i2}-\left( 1-s_{2} \right)c_{2}$, are equivalent since if person $i$ gets vaccinated with vaccine 2, then it is irrelevant whether vaccine 1 is also produced. Analogously, lines 4 and 6, causing utility $V_{i1}-\left( 1-s_{1} \right)c_{1}$, are equivalent since if person $i$ gets vaccinated with vaccine 1, then it is irrelevant whether vaccine 2 is also produced. Lines 7,8,10,12 in (9) are equivalent since if person $i$ does not buy any drugs, then it is irrelevant whether the drug is produced or not, and irrelevant whether drug 1 or drug 2 or both drugs are available. The equivalent lines 9 and 13 state that person$i$ buys drug 2 at cost $\left( 1-S_{2} \right)C_{2}$. The equivalent lines 11 and 14 state that person $i$ buys drug 1 at cost $\left( 1-S_{1} \right)C_{1}$.

When no drugs are produced, only outcomes 1-7 in (9) are possible. Outcome 3 causes death with probability $1-x$ and recovery with probability $x$. Hence all the six utilities $H_{i},\left( 1-q\left( \left( m_{1}\left( t \right)+m_{2}\left( t \right) \right)/N \right) \right)^{rn}E_{i},V_{ik},D_{i},R_{i}$, $k=1,2$, are possible. If only drug company 1 produces the drug, outcomes 1,2,10,11 in (9) are possible. If only drug company 2 produces the drug, outcomes 1,2,8,9 in (9) are possible. If both drug companies produce the drug, outcomes 1,2,12,13,14 in (9) are possible. The $N$ persons differ in their utilities $H_{i},\left( 1-q\left( \left( m_{1}\left( t \right)+m_{2}\left( t \right) \right)/N \right) \right)^{rn}E_{i},V_{ik},D_{i},R_{i}$, and disperse across the 14 outcomes depending on whether no drugs, drug 1 or drug 2, or both drugs are produced, and depending on whether no vaccines, vaccine 1 or vaccine 2, or both vaccines are produced.

Summing up, person$i$ has minimally one, more commonly three, and sometimes more than three strategic choice variables. It inevitably chooses risky or safe behavior in period 1 in Figure 2, which leads person $i$ either to the vaccination game in Figure 3 or the drug game in Figure 4. If Figure 3 is reached and vaccine company $k$ produces vaccine $k$, $k=1,2$, person $i$ chooses whether or not to buy vaccine $k$ in period 2 in Figure 3, which gives two additional strategies. Alternatively, if Figure 4 is reached and drug company $j$ produces drug $j, j=1,2$, person $i$ chooses whether or not to buy drug $j$ in period 2 in Figure 4, which also gives two additional strategic choices. It is theoretically possible that person $i$ makes two strategic choices not to buy vaccine $k$ in Figure 3, causing return to period 1 in Figure 2 where person $i$ chooses risky behavior, causing subsequent transitions to Figure 3 or Figure 4 to make additional strategic choices.

### *B4 Donor’s behavior, benefits, and costs*

The donor’s benefit is the sum of the $N$ persons’ benefits $H_{i},E_{i},V_{ik},D_{i},R_{i}$ (ignoring the $N$ persons’ purchasing costs of vaccines and drugs), spread across the 14 outcomes in Figure 2, Figure 3, and Figure 4, dispersed across the seven groups in Figure 5, accounting for Nature’s probabilities $q\left( \left( m_{1}\left( t \right)+m_{2}\left( t \right) \right)/N \right),x,w_{j}$, and subtracting the donor’s cost of subsidy choices of $y_{k}$,$s_{k}$,$Y_{j}$,$S_{j}$ where $m_{k}=\lim_{t\to\infty} m_{k}\left( t \right)$. The donor’s expected utility is

| $V=\left\{ \begin{matrix} \sum_{i=1}^{G} H_{i}+\sum_{i=G+1}^{G+L-m_{2}-m_{1}} \left( 1-q\left( \frac{m_{1}+m_{2}}{N} \right) \right)^{rn}E_{i}+\sum_{i=G+L-m_{2}-m_{1}+1}^{G+L-m_{1}} V_{i2} \\ -I_{v2}y_{2}f_{2}-m_{2}s_{2}c_{2}+\sum_{i=G+L-m_{1}+1}^{G+L} V_{i1}-I_{v1}y_{1}f_{1}-m_{1}s_{1}c_{1} \\ \begin{matrix} +\sum_{i=G+L+1}^{N-M_{2}-M_{1}} \left[ \left( 1-x \right)D_{i}+xR_{i} \right]+\sum_{i=N-M_{2}-M_{1}+1}^{N-M_{1}} \left[ \left( 1-w_{2} \right)D_{i}+w_{2}R_{i} \right] \\ -I_{d2}Y_{2}F_{2}-M_{2}S_{2}C_{2}+\sum_{i=N-M_{1}+1}^{N} \left[ \left( 1-w_{1} \right)D_{i}+w_{1}R_{i} \right]-I_{d1}Y_{1}F_{1}-M_{1}S_{1}C_{1} \end{matrix} \end{matrix} \right.$ | (10) |
| --- | --- |

where the seven summation signs correspond to the seven groups in Figure 5. Summation sign 1 is for the $G$ persons choosing safe behavior. Summation sign 2 is for the $L-m_{2}-m_{1}$ persons choosing risky behavior while not contracting the disease and not purchasing vaccines. Summation signs 3 and 4 are for the $m_{2}$ and $m_{1}$ persons buying vaccines 1 and 2, respectively. Summation sign 5 is for the $N-G-L-M_{2}-M_{1}$ persons contracting the disease and not buying drugs. Summation signs 6 and 7 are for the $M_{2}$ and $M_{1}$ persons buying drugs 1 and 2, respectively. The two subtractions after summation signs 3,4,6,7 are the donor’s cost of subsidy choices of $y_{k}$,$s_{k}$,$Y_{j}$,$S_{j}$.

The subtraction $I_{vk}y_{k}f_{k}$ after summation signs 3 and 4 are the donor’s subsidy fraction $y_{k}$ of vaccine company $k$’s development cost $f_{k}$, where the indicator parameter $I_{vk}$ equals 1 if vaccine $k$ is produced, and equals 0 otherwise, $k=1,2$. The subtraction $m_{k}s_{k}c_{k}$ after summation signs 3 and 4 are the donor’s subsidy fraction $s_{k}$ of person $i$’s vaccine $k$’s purchasing cost $c_{k}$, multiplied with the number $m_{k}$ of persons purchasing vaccine $k$, $k=1,2$.

Analogously, the subtraction $I_{dj}Y_{j}F_{j}$ after summation signs 6 and 7 are the donor’s subsidy fraction $Y_{j}$ of drug company $j$’s development cost $F_{j}$, where the indicator parameter $I_{dj}$ equals 1 if drug $j$ is produced, and equals 0 otherwise, $j=1,2$. The subtraction $M_{j}S_{j}C_{j}$ after summation signs 6 and 7 are the donor’s subsidy fraction $S_{j}$ of person $i$’s drug $j$’s purchasing cost $C_{j}$, multiplied with the number $M_{j}$ of persons purchasing drug $j$, $j=1,2$.

Summing up, the donor has eight strategic choice variables. It chooses whether to subsidize a fraction $y_{k}$ for vaccine $k$ development in period 1 in Figure 3, whether to subsidize a fraction $s_{k}$ for each person $i$’s vaccine $k$ purchase in period 2 in Figure 3, $k=1,2$. It chooses whether to subsidize a fraction $Y_{j}$ for drug $j$ development in period 1 in Figure 4, and whether to subsidize a fraction $S_{j}$ for each person $i$’s drug $j$ purchase in period 2 in Figure 4, $j=1,2$.

### *B5 Nature*

Nature has eight strategic choice variables. It chooses the disease contraction probability $q\left( \left( m_{1}\left( t \right)+m_{2}\left( t \right) \right)/N \right)$ in period 2 in Figure 2, the recovery probability $w_{j}$ in period 3 in Figure 4 if the drug is bought (and applied), and the recovery probability $x$ in period 3 in Figure 4 if no drug is produced or purchased, $0\leq x\leq w_{j}\leq1$, $j=1,2$. Nature also chooses, at the same time and given that the companies choose to develop vaccines and drugs, whether vaccines and drugs are developed successfully.
