# Supplementary material for: A Game Theoretic Analysis of Competition Between Vaccine and Drug Companies during Disease Contraction and Recovery
Source: Med Decis Making. 2021 Nov 5;42(5):571–86. doi: 10.1177/0272989X211053563 (PMC9189729; doi:10.1177/0272989X211053563)
Supplement: sj-docx-3-mdm-10.1177_0272989X211053563 – Supplemental material for A Game Theoretic Analysis of Competition Between Vaccine and Drug Companies during Disease Contraction and Recovery [file sj-docx-3-mdm-10.1177_0272989X211053563.docx]

Appendix C Analyzing the model

The game is solved with backward induction.

### *C1 Period 3 in Figure 4: Nature*

In period 3 in Figure 4 Nature chooses the disease recovery with the probabilities $w_{j}$ and $x$, when drug $j$ has been applied and not applied, respectively, $j=1,2$.

### *C2 Period 2 in Figure 4 and Figure 3: Person* $i$ *and donor*

#### C2.1 Person $i$

Applying (9), in period 2 in Figure 4 person $i$ buys drug 1 if the benefits outweigh those of buying drug 2 or not buying drugs, that is, if

| $\left( 1-w_{1} \right)D_{i}+w_{1}R_{i}-\left( 1-S_{1} \right)C_{1}\geq Max\left( \begin{aligned} \left( 1-w_{2} \right)D_{i}+w_{2}R_{i}-\left( 1-S_{2} \right)C_{2}, \\ \left( 1-x \right)D_{i}+xR_{i} \end{aligned} \right)$ | (11) |
| --- | --- |

Analogously, person $i$ buys drug 2 if

| $\left( 1-w_{2} \right)D_{i}+w_{2}R_{i}-\left( 1-S_{2} \right)C_{2}\geq Max\left( \begin{aligned} \left( 1-w_{1} \right)D_{i}+w_{1}R_{i}-\left( 1-S_{1} \right)C_{1}, \\ \left( 1-x \right)D_{i}+xR_{i} \end{aligned} \right)$ | (12) |
| --- | --- |

and buys no drugs if

| $\left( 1-x \right)D_{i}+xR_{i}\geq Max\left( \begin{aligned} \left( 1-w_{1} \right)D_{i}+w_{1}R_{i}-\left( 1-S_{1} \right)C_{1}, \\ \left( 1-w_{2} \right)D_{i}+w_{2}R_{i}-\left( 1-S_{2} \right)C_{2} \end{aligned} \right)$ | (13) |
| --- | --- |

Analogously, and also applying (9), in period 2 in Figure 3 person $i$ buys vaccine 1 if the benefits outweigh those of buying vaccine 2 or not buying vaccines, that is

| $V_{i1}-\left( 1-s_{1} \right)c_{1}\geq Max\left( V_{i2}-\left( 1-s_{2} \right)c_{2},\left( 1-q\left( \left( m_{1}\left( t \right)+m_{2}\left( t \right) \right)/N \right) \right)^{rn}E_{i} \right)$ | (14) |
| --- | --- |

Analogously, person $i$ buys vaccine 2 if

| $V_{i2}-\left( 1-s_{2} \right)c_{2}\geq Max\left( V_{i1}-\left( 1-s_{1} \right)c_{1},\left( 1-q\left( \left( m_{1}\left( t \right)+m_{2}\left( t \right) \right)/N \right) \right)^{rn}E_{i} \right)$ | (15) |
| --- | --- |

and buys no vaccines if

| $\left( 1-q\left( \left( m_{1}\left( t \right)+m_{2}\left( t \right) \right)/N \right) \right)^{rn}E_{i}\geq Max\left( V_{i1}-\left( 1-s_{1} \right)c_{1},V_{i2}-\left( 1-s_{2} \right)c_{2} \right)$ | (16) |
| --- | --- |

Property 1. Person $i$ prefers drug $j$ rather than drug $g$, $j,g=1,2, j\neq g$, if the disease recovery probability $w_{j}$ with drug $j$ is high compared with $w_{g}$ with drug $g$, if drug $j$ has low cost $C_{j}$ compared with $C_{g}$ for drug $g$, and if the donor subsidy fraction $S_{j}$ for buying drug $j$ is high compared with the subsidy fraction $S_{g}$ for buying drug $g$. Otherwise person $i$ prefers drug $g$.

Proof. Follows from (11), (12), and ${0>D}_{i}<R_{i}>0$.

In Property 1, and the properties below, “high” or “low” “compared with” does not mean “higher” or “lower” “than” for all the instances mentioned. However, with the weights $D_{i}$ and $R_{i}$ in (11) and (12), “high” or “low” “compared with” must be satisfied sufficiently often such that the inequalities in (11) and (12) are satisfied. The various if-conditions within each property may pull in opposite directions, or may be challenging to satisfy simultaneously. For example, for Property 1, a drug $j$ with high disease recovery probability $w_{j}$ may be challenging to reconcile with low cost $C_{j}$, unless the donor subsidizes substantially with high $S_{j}$. That illustrates the delicate balance that has to be struck by the players.

Property 2. Person $i$ prefers drug $j$, $j=1,2$, rather than no drug if the disease recovery probability $w_{j}$ with drug $j$ is high compared with $x$ with no drug, if drug $j$ has low cost $C_{j}$, and if the donor subsidy fraction $S_{j}$ for buying drug $j$ is high. Otherwise person $i$ prefers no drug.

Proof. Follows from (13).

Property 3. Person $i$ prefers vaccine $k$ rather than vaccine $h$, $k,h=1,2, k\neq h$, if its utility $V_{ik}$ of vaccine $k$ is high compared with $V_{ih}$ with vaccine $h$, if vaccine $k$ has low cost $c_{k}$ compared with $c_{h}$ for vaccine $h$, and if the donor subsidy fraction $s_{k}$ for buying vaccine $k$ is high compared with the subsidy fraction $s_{h}$ for buying vaccine $h$. Otherwise person $i$ prefers vaccine $h$.

Proof. Follows from (14) and (15).

In Property 3, a vaccine $k$ with high utility $V_{ik}$ may be challenging to reconcile with low cost $c_{k}$, unless the donor subsidizes substantially with high $s_{k}$.

Property 4. Person $i$ prefers vaccine $k$, $j=1,2$, rather than no vaccine if its utility $V_{ik}$ of vaccine $k$ is high compared with $\left( 1-q\left( \left( m_{1}\left( t \right)+m_{2}\left( t \right) \right)/N \right) \right)^{rn}E_{i}$ with no vaccine, if vaccine $k$ has low cost $c_{k}$, and if the donor subsidy fraction $s_{k}$ for buying vaccine $k$ is high. Otherwise person $i$ prefers no vaccine.

Proof. Follows from (16).

In Property 4, $E_{i}$ is person $i$’s utility of risky behavior, $1-q\left( \left( m_{1}\left( t \right)+m_{2}\left( t \right) \right)/N \right)$ is the probability of not contracting the disease at time $t$, and $n$ is the number of returns from period 2 in Figure 3 to period 1 in Figure 2 when assessing $E_{i}$, with $r$ as a scaling parameter.

#### C2.2 Donor

In period 2 in Figure 4 the donor chooses whether to subsidize a fraction $S_{j}$ of person $i$’s drug $j$ purchasing cost $C_{j}$ to maximize its expected utility $V$ in (10). The donor must thus justify the cost $M_{1}S_{1}C_{1}+M_{2}S_{2}C_{2}$ to the $M_{1}$ persons buying drug 1 and the $M_{2}$ persons buying drug 2. Assume that donor sponsoring is imperative for the $M_{1}+M_{2}$ persons to buy drugs, and that they would otherwise not buy drugs. When $M_{1}+M_{2}$ drugs are bought, summation signs 6 and 7 are as in (10). When $M_{1}+M_{2}$ drugs are not bought, summation signs 6 and 7 are as in (10) when replacing the high disease recovery probability $w_{j}$ with drug $j$ with the low disease recovery probability $x$ without drug, $j=1,2$. Summation signs 6 and 7 in (10) are higher with $w_{j}$ than with $x$, since $x\leq w_{j}$ and ${0>D}_{i}<R_{i}>0$. That is, the $M_{1}+M_{2}$ drugs causes fewer deaths. Using (10) to express this mathematically, the donor sponsors person $i$’s drug $j$ purchasing cost $C_{j}$ with the subsidy fraction $S_{j}$ when

| $\begin{matrix} \sum_{i=N-M_{2}-M_{1}+1}^{N-M_{1}} \left[ \left( 1-w_{2} \right)D_{i}+w_{2}R_{i} \right]-\sum_{i=N-M_{2}-M_{1}+1}^{N-M_{1}} \left[ \left( 1-x \right)D_{i}+xR_{i} \right]-M_{2}S_{2}C_{2} \\ +\sum_{i=N-M_{1}+1}^{N} \left[ \left( 1-w_{1} \right)D_{i}+w_{1}R_{i} \right]-\sum_{i=N-M_{1}+1}^{N} \left[ \left( 1-x \right)D_{i}+xR_{i} \right]-M_{1}S_{1}C_{1}\geq0 \end{matrix}$ | (17) |
| --- | --- |

Analogously, in period 2 in Figure 3 the donor chooses whether to subsidize a fraction $s_{k}$ of person $i$’s vaccine $k$ purchasing cost $c_{k}$ to maximize its expected utility $V$ in (10). Analogously to the reasoning for drug $j$, the donor must justify the cost $m_{1}s_{1}c_{1}+m_{2}s_{2}c_{2}$ to the $m_{1}$ persons buying vaccine 1 and the $m_{2}$ persons buying vaccine 2. Assume that donor sponsoring is imperative for the $m_{1}+m_{2}$ persons to buy vaccines, and that they would otherwise not buy vaccines. Hence the low disease contraction probability $q\left( \frac{m_{1}+m_{2}}{N} \right)$ in summation sign 2 in (10) is replaced with the high disease contraction probability $q\left( 0 \right)$, and person $i$’s utility $V_{ik}$ of vaccine $k$ vaccination in summation signs 3 and 4 in (10) is replaced with the higher utility $E_{i}$ of risky behavior, $V_{ik}<E_{i}$, $k=1,2$. Using (10) to express this mathematically, the donor sponsors person $i$’s vaccine $k$ purchasing cost $c_{k}$ with the subsidy fraction $s_{k}$ when

| $\begin{matrix} \sum_{i=G+1}^{G+L-m_{2}-m_{1}} \left( 1-q\left( \frac{m_{1}+m_{2}}{N} \right) \right)^{rn}E_{i}-\sum_{i=G+1}^{G+L-m_{2}-m_{1}} \left( 1-q\left( 0 \right) \right)^{rn}E_{i} \\ +\sum_{i=G+L-m_{2}-m_{1}+1}^{G+L-m_{1}} \left( V_{i2}-E_{i} \right)-m_{2}s_{2}c_{2}+\sum_{i=G+L-m_{1}+1}^{G+L} \left( V_{i1}-E_{i} \right)-m_{1}s_{1}c_{1}\geq0 \end{matrix}$ | (18) |
| --- | --- |

### *C3 Period 1 in Figure 4 and Figure 3: Drug company* $j$*, vaccine company* $k$*, donor*

#### C3.1 Drug company $j$

Applying (8), in period 1 in Figure 4 drug company $j$ develops drug $j$, sponsored by a donor, if it is profitable, that is, if

| $g_{dj}\left[ M_{j}C_{j}-\left( M_{j}B_{j} \right)^{A_{j}} \right]-\left( 1-Y_{j} \right)F_{j}\geq0$ | (19) |
| --- | --- |

Property 5. Drug company $j$, $j=1,2$, develops drug $j$ if many persons $M_{j}$ buy drug $j$ at a high price $C_{j}$, if the drug $j$ production cost $B_{j}$ destined for the $M_{j}$ persons accounting for the scaling $A_{j}$ of production is low, if the drug $j$ development cost $F_{j}$ is low, if Nature’s probability $g_{dj}$ is high that drug $j$ is developed successfully, and if the donor’s subsidy fraction $Y_{j}$ of the drug $j$ development cost $F_{j}$ is high.

Proof. Follows from (19).

Also in Property 5, the various if-conditions may pull in opposite directions, or may be challenging to satisfy simultaneously. For example, for Property 5, it may be challenging to get many persons $M_{j}$ to buy drug $j$ at a high price $C_{j}$, if at the same time the drug $j$ production cost $B_{j}$, the drug $j$ development cost $F_{j}$, and the donor’s subsidy fraction $Y_{j}$ of the drug $j$ development cost $F_{j}$, are low. This again illustrates the delicate balance that has to be struck by the players.

#### C3.2 Vaccine company $k$

Applying (7), in period 1 in Figure 3 vaccine company $k$ develops vaccine $k$, sponsored by a donor, if it is profitable, that is, if

| $g_{vk}\left[ m_{k}c_{k}-\left( m_{k}b_{k} \right)^{a_{k}} \right]-\left( 1-y_{k} \right)f_{k}\geq0$ | (20) |
| --- | --- |

Property 6. Vaccine company $k$, $k=1,2$, develops vaccine $k$ if many persons $m_{k}$ buy vaccine $k$ at a high price $c_{k}$, if the vaccine $k$ production cost $b_{k}$ destined for the $m_{k}$ persons accounting for the scaling $a_{k}$ of production is low, if the vaccine $k$ development cost $f_{k}$ is low, if Nature’s probability $g_{vk}$ is high that vaccine $k$ is developed successfully, and if the donor’s subsidy fraction $y_{k}$ of the vaccine $k$ development cost $f_{k}$ is high.

Proof. Follows from (20).

Property 6 for vaccine company $k$ is analogous to Property 5 for drug company $j$, where the corresponding if-conditions may pull in opposite directions.

#### C3.3 Donor

In period 1 in Figure 4 the donor chooses whether to subsidize a fraction $Y_{j}$ of the drug $j$ development cost $F_{j}$ to maximize its expected utility $V$ in (10). The donor must thus justify the cost $I_{d1}Y_{1}F_{1}+I_{d2}Y_{2}F_{2}$ to the two drug companies of producing drugs. Assume that donor sponsoring is imperative for the two drug companies to produce drugs, and that they would otherwise not produce drugs. When the two drugs are produced, summation signs 5, 6 and 7 are as in (10). When the two drugs are not produced, they also cannot be bought. Hence $M_{1}=M_{2}=0$, causing summation signs 6 and 7 in (10) to disappear, and summation sign 5 to run from $i=G+L+1$ to $i=N$. Using (10) to express this mathematically, the donor sponsors the drug $j$ development cost $F_{j}$ with the subsidy fraction $Y_{j}$ when

| $\begin{matrix} \sum_{i=G+L+1}^{N-M_{2}-M_{1}} \left[ \left( 1-x \right)D_{i}+xR_{i} \right]+\sum_{i=N-M_{2}-M_{1}+1}^{N-M_{1}} \left[ \left( 1-w_{2} \right)D_{i}+w_{2}R_{i} \right] \\ -I_{d2}Y_{2}F_{2}+\sum_{i=N-M_{1}+1}^{N} \left[ \left( 1-w_{1} \right)D_{i}+w_{1}R_{i} \right]-I_{d1}Y_{1}F_{1}-\sum_{i=G+L+1}^{N} \left[ \left( 1-x \right)D_{i}+xR_{i} \right]\geq0 \end{matrix}$ | (21) |
| --- | --- |

Analogously, in period 1 in Figure 3 the donor chooses whether to subsidize a fraction $y_{k}$ of the vaccine $k$ development cost $f_{k}$ to maximize its expected utility $V$ in (10). Analogously to the reasoning for drug $j$, the donor must justify the cost $I_{v1}y_{1}f_{1}+I_{v2}y_{2}f_{2}$ to the two vaccine companies of producing vaccines. Assume that donor sponsoring is imperative for the two vaccine companies to produce vaccines, and that they would otherwise not produce vaccines. When the two vaccines are produced, summation signs 2, 3 and 4 are as in (10). When the two vaccines are not produced, they also cannot be bought. Hence $m_{1}=m_{2}=0$, causing summation signs 3 and 4 in (10) to disappear, and summation sign 2 to run from $i=G+1$ to $i=G+L$, replacing the low disease contraction probability $q\left( \frac{m_{1}+m_{2}}{N} \right)$ with the high disease contraction probability $q\left( 0 \right)$. Using (10) to express this mathematically, the donor sponsors the vaccine $k$ development cost $f_{k}$ with the subsidy fraction $y_{k}$ when

| $\begin{matrix} \sum_{i=G+1}^{G+L-m_{2}-m_{1}} \left( 1-q\left( \frac{m_{1}+m_{2}}{N} \right) \right)^{rn}E_{i}+\sum_{i=G+L-m_{2}-m_{1}+1}^{G+L-m_{1}} V_{i2} \\ -I_{v2}y_{2}f_{2}+\sum_{i=G+L-m_{1}+1}^{G+L} V_{i1}-I_{v1}y_{1}f_{1}-\sum_{i=G+1}^{G+L} \left( 1-q\left( 0 \right) \right)^{rn}E_{i}\geq0 \end{matrix}$ | (22) |
| --- | --- |

### *C4 Period 2 in Figure 2: Nature*

In period 2 in Figure 2 Nature chooses disease contraction with the probability $q\left( \left( m_{1}\left( t \right)+m_{2}\left( t \right) \right)/N \right)$, and hence the disease is not contracted with probability $1-q\left( \left( m_{1}\left( t \right)+m_{2}\left( t \right) \right)/N \right)$.

### *C5 Period 1 in Figure 2: Person* $i$

In period 1 in Figure 2 person $i$ chooses risky behavior with endogenous probability $p$, and risky behavior with endogenous probability $1-p$.

Property 7. If neither drug $j, j=1,2$, nor vaccine $k, k=1,2$, are optimal for person $i$ to buy, it chooses risky behavior if

| $\left( 1-q\left( \left( m_{1}\left( t \right)+m_{2}\left( t \right) \right)/N \right) \right)^{1+rn}E_{i}+q\left( \left( m_{1}\left( t \right)+m_{2}\left( t \right) \right)/N \right)\left( \left( 1-x \right)D_{i}+xR_{i} \right)\geq H_{i}$ | (23) |
| --- | --- |

Proof. Follows from (9).

Property 7 states that person $i$ chooses risky behavior if person $i$’s utility $E_{i}$ of risky behavior, person $i$’s utility $R_{i}$ when recovering from the disease, and person $i$’s disease recovery probability $x$ without drug $j$, are high; when the negative utility $D_{i}$ of death is low in absolute value; and when person $i$’s utility $H_{i}$ of safe behavior, person $i$’s disease contraction probability $q\left( \left( m_{1}\left( t \right)+m_{2}\left( t \right) \right)/N \right)$, the number $n$ of returns from period 2 in Figure 3 to period 1 in Figure 2 when assessing $E_{i}$, and the scaling parameter $r$ for $n$, are low.

Property 8. If drug $j, j=1,2$, is not optimal for person $i$ to buy, but vaccine $k, k=1,2$, is optimal for person i to buy, it chooses risky behavior if

| $\left( 1-q\left( \left( m_{1}\left( t \right)+m_{2}\left( t \right) \right)/N \right) \right)\left( V_{ik}-\left( 1-s_{k} \right)c_{k} \right)+q\left( \left( m_{1}\left( t \right)+m_{2}\left( t \right) \right)/N \right)\left( \left( 1-x \right)D_{i}+xR_{i} \right)\geq H_{i}$ | (24) |
| --- | --- |

Proof. Follows from (9).

Property 8 states that person $i$ chooses risky behavior if person $i$’s utility $V_{ik}$ of vaccine $k$ vaccination, the donor’s subsidy fraction $s_{k}$ of person $i$’s vaccine $k$ purchasing cost $c_{k}$, person $i$’s utility $R_{i}$ when recovering from the disease, and person $i$’s disease recovery probability $x$ without drug $j$, are high; when the negative utility $D_{i}$ of death is low in absolute value; and when person $i$’s vaccine $k$ purchasing cost $c_{k}$, person $i$’s utility $H_{i}$ of safe behavior, and person $i$’s disease contraction probability $q\left( \left( m_{1}\left( t \right)+m_{2}\left( t \right) \right)/N \right)$, are low.

Property 9. If drug $j, j=1,2$, is optimal for person $i$ to buy, but vaccine $k, k=1,2$, is not optimal for person i to buy, it chooses risky behavior if

| $\left( 1-q\left( \left( m_{1}\left( t \right)+m_{2}\left( t \right) \right)/N \right) \right)^{1+rn}E_{i}+q\left( \left( m_{1}\left( t \right)+m_{2}\left( t \right) \right)/N \right)\left( \left( 1-w_{j} \right)D_{i}+w_{j}R_{i}-\left( 1-S_{j} \right)C_{j} \right)\geq H_{i}$ | (25) |
| --- | --- |

Proof. Follows from (9).

Property 9 states that person $i$ chooses risky behavior if person $i$’s utility $E_{i}$ of risky behavior, person $i$’s utility $R_{i}$ when recovering from the disease, person $i$’s disease recovery probability $w_{j}$ with drug $j$, and the donor’s subsidy fraction $S_{j}$ of person $i$’s drug $j$ purchasing cost $C_{j}$, are high; when the negative utility $D_{i}$ of death is low in absolute value; and when person $i$’s drug $j$ purchasing cost $C_{j}$, person $i$’s utility $H_{i}$ of safe behavior, person $i$’s disease contraction probability $q\left( \left( m_{1}\left( t \right)+m_{2}\left( t \right) \right)/N \right)$, the number $n$ of returns from period 2 in Figure 3 to period 1 in Figure 2 when assessing $E_{i}$, and the scaling parameter $r$ for $n$, are low.

Property 10. If both drug $j, j=1,2$, and vaccine $k, k=1,2$, are optimal for person $i$ to buy, it chooses risky behavior if

| $\left( 1-q \right)\left( V_{ik}-\left( 1-s_{k} \right)c_{k} \right)+q\left( \left( 1-w_{j} \right)D_{i}+w_{j}R_{i}-\left( 1-S_{j} \right)C_{j} \right)\geq H_{i}$ | (26) |
| --- | --- |

Proof. Follows from (9).

Property 10 states that person $i$ chooses risky behavior if person $i$’s utility $V_{ik}$ of vaccine $k$ vaccination, the donor’s subsidy fraction $s_{k}$ of person $i$’s vaccine $k$ purchasing cost $c_{k}$, person $i$’s utility $R_{i}$ when recovering from the disease, person $i$’s disease recovery probability $w_{j}$ with drug $j$, and the donor’s subsidy fraction $S_{j}$ of person $i$’s drug $j$ purchasing cost $C_{j}$, are high; when the negative utility $D_{i}$ of death is low in absolute value; and when person $i$’s drug $j$ purchasing cost $C_{j}$, person $i$’s vaccine $k$ purchasing cost $c_{k}$, person $i$’s utility $H_{i}$ of safe behavior, and person $i$’s disease contraction probability $q\left( \left( m_{1}\left( t \right)+m_{2}\left( t \right) \right)/N \right)$, are low.
