# Supplementary material for: A Game Theoretic Analysis of Competition Between Vaccine and Drug Companies during Disease Contraction and Recovery
Source: Med Decis Making. 2021 Nov 5;42(5):571–86. doi: 10.1177/0272989X211053563 (PMC9189729; doi:10.1177/0272989X211053563)
Supplement: sj-docx-4-mdm-10.1177_0272989X211053563 – Supplemental material for A Game Theoretic Analysis of Competition Between Vaccine and Drug Companies during Disease Contraction and Recovery [file sj-docx-4-mdm-10.1177_0272989X211053563.docx]

**Appendix D The 14 outcomes**

Table 1 lists the 14 outcomes O, which are the 14 lines in (9), in the left column, the description in column 2, the equations that apply in column 3, the conditions in the numerical example in Section 3.2 in column 4, and the results for the numerical example in the right column. These 14 outcomes account for all the possible combinations of all the strategies by all the players, i.e. person $i$, $i=1,\ldots,N$, the two vaccine companies, the two drug companies, the donor, and Nature.

Table 1 The 14 outcomes O, the description, the equations that apply, the conditions for the numerical example in Section 3.2, and the results for the numerical example.

| O | Description | Equations | Conditions in numerical example | Result for numerical example |
| --- | --- | --- | --- | --- |
| 1 | Person $i$ prefers safe behavior | (23),(24),(25),(26) are not satisfied. | (23):$604100(n=3)\geq5\times{10}^{5}$  (24)$k=1:667991\geq5\times{10}^{5}$  (24)$k=2:667991\geq5\times{10}^{5}$  (25)$j=1:640098\geq5\times{10}^{5}$  (25)$j=2:640058\geq5\times{10}^{5}$  (26)$j=k=1:703989\geq5\times{10}^{5}$  (26)$j=1,k=2:703989\geq5\times{10}^{5}$  (26)$j=2,k=1:703949\geq5\times{10}^{5}$  (26)$j=k=2:703949\geq5\times{10}^{5}$ | Impossible; person $i$ prefers risky behavior |
| 2 | No disease contraction & no vaccination, causing risky behavior | (20) is not satisfied for either $k=1$ or $k=2$. If (20) is satisfied, then (14) and (15) are not satisfied. (23) or (25) is satisfied | (14):$799990\geq799990$  (15):$799990\geq799990$  (18):$3.58\times{10}^{12}\geq0$  (20)$g_{vk}=1,k=1: 3.35\times{10}^{8}\geq0$  (20)$g_{vk}=1,k=2: 3.35\times{10}^{8}\geq0$  (22):$2.99\times{10}^{13}\geq0$  (23):$604100(n=3)\geq5\times{10}^{5}$  (25)$j=1:640098\geq5\times{10}^{5}$  (25)$j=2:640058\geq5\times{10}^{5}$ | Impossible; person $i$ buys either vaccine or vaccine 2; both (14) and (15) are satisfied |
| 3 | No disease contraction & vaccine 2 production & vaccination | (20) is not satisfied when $k=1$, (20) is satisfied when $k=2$; (15) and (24) are satisfied | (15):$799990\geq799990$  (18):$3.58\times{10}^{12}\geq0$  (20)$g_{vk}=1,k=1: 3.35\times{10}^{8}\geq0$  (20)$g_{vk}=1,k=2: 3.35\times{10}^{8}\geq0$  (22):$2.99\times{10}^{13}\geq0$  (24)$k=1:667991\geq5\times{10}^{5}$  (24)$k=2:667991\geq5\times{10}^{5}$ | Impossible; vaccine 1 is produced; (20) and (22) are always satisfied |
| 4 | No disease contraction & vaccine 1 production & vaccination | (20) is satisfied when $k=1$, (20) is not satisfied when $k=2$;(14) and (24) are satisfied | (14):$799990\geq799990$  (18):$3.58\times{10}^{12}\geq0$  (20)$g_{vk}=1,k=1: 3.35\times{10}^{8}\geq0$  (20)$g_{vk}=1,k=2: 3.35\times{10}^{8}\geq0$  (22):$2.99\times{10}^{13}\geq0$  (24)$k=1:667991\geq5\times{10}^{5}$  (24)$k=2:667991\geq5\times{10}^{5}$ | Impossible; vaccine 2 is produced; (20) and (22) are always satisfied |
| 5 | No disease contraction & vaccines 1&2 production & vaccine 2 vaccination | (15),(20),(24) are satisfied | (15):$799990\geq799990$  (18):$3.58\times{10}^{12}\geq0$  (20)$g_{vk}=1,k=1: 3.35\times{10}^{8}\geq0$  (20)$g_{vk}=1,k=2: 3.35\times{10}^{8}\geq0$  (22):$2.99\times{10}^{13}\geq0$  (24)$k=1:667991\geq5\times{10}^{5}$  (24)$k=2:667991\geq5\times{10}^{5}$ | Possible; person $i$ buys either vaccine 1 or vaccine 2, but not both |
| 6 | No disease contraction & vaccines 1&2 production & vaccine 1 vaccination | (14),(20),(24) are satisfied | (14):$799990\geq799990$  (18):$3.58\times{10}^{12}\geq0$  (20)$g_{vk}=1,k=1: 3.35\times{10}^{8}\geq0$  (20)$g_{vk}=1,k=2: 3.35\times{10}^{8}\geq0$  (22):$2.99\times{10}^{13}\geq0$  (24)$k=1:667991\geq5\times{10}^{5}$  (24)$k=2:667991\geq5\times{10}^{5}$ | Possible; person $i$ buys either vaccine 1 or vaccine 2, but not both |
| 7 | Disease contraction & no drug production | (19) is not satisfied; (23) or (24) or (25) or (26) is satisfied. | (17):1$.44\times{10}^{13}\geq0$  (19)$g_{dj}=1,j=1:6.49\times{10}^{10}\geq0$  (19)$g_{dj}=1,j=2:1.42\times{10}^{10}\geq0$  (21):1$.44\times{10}^{13}\geq0$  (23):$604100(n=3)\geq5\times{10}^{5}$  (24)$k=1:667991\geq5\times{10}^{5}$  (24)$k=2:667991\geq5\times{10}^{5}$  (25)$j=1:640098\geq5\times{10}^{5}$  (25)$j=2:640058\geq5\times{10}^{5}$  (26)$j=k=1:703989\geq5\times{10}^{5}$  (26)$j=1,k=2:703989\geq5\times{10}^{5}$  (26)$j=2,k=1:703949\geq5\times{10}^{5}$  (26)$j=k=2:703949\geq5\times{10}^{5}$ | Impossible; (19) and (21) are satisfied and hence both drugs are produced |
| 8 | Disease contraction with drug production, but without buying drug 2 | (12) is not satisfied; (19) is not satisfied when $j=1$, (19) is satisfied when $j=2;$(23) and (24) are satisfied. | (12):$-160425<-160019$  (17):1$.44\times{10}^{13}\geq0$  (19)$g_{dj}=1,j=1:6.49\times{10}^{10}\geq0$  (19)$g_{dj}=1,j=2:1.42\times{10}^{10}\geq0$  (21):1$.44\times{10}^{13}\geq0$  (23):$604100(n=3)\geq5\times{10}^{5}$  (24)$k=1:667991\geq5\times{10}^{5}$  (24)$k=2:667991\geq5\times{10}^{5}$ | Impossible; drug 1 is produced; (19) and (21) are always satisfied |
| 9 | Disease contraction with drug production and buying drug 2 | (12) is satisfied; (19) is not satisfied when $j=1$, (19) is satisfied when $j=2;$  (25) or (26) is satisfied. | (12):$-160425<-160019$  (17):1$.44\times{10}^{13}\geq0$  (19)$g_{dj}=1,j=1:6.49\times{10}^{10}\geq0$  (19)$g_{dj}=1,j=2:1.42\times{10}^{10}\geq0$  (21):1$.44\times{10}^{13}\geq0$  (25)$j=1:640098\geq5\times{10}^{5}$  (25)$j=2:640058\geq5\times{10}^{5}$  (26)$j=k=1:703989\geq5\times{10}^{5}$  (26)$j=1,k=2:703989\geq5\times{10}^{5}$  (26)$j=2,k=1:703949\geq5\times{10}^{5}$  (26)$j=k=2:703949\geq5\times{10}^{5}$ | Impossible; drug 1 is produced; (19) and (21) are always satisfied |
| 10 | Disease contraction with drug production, but without buying drug 1 | (11) is not satisfied; (19) is satisfied when $j=1$, (19) is not satisfied when $j=2$; (23) and (24) are satisfied. | (11):$-160019\geq-160425$  (17):1$.44\times{10}^{13}\geq0$  (19)$g_{dj}=1,j=1:6.49\times{10}^{10}\geq0$  (19)$g_{dj}=1,j=2:1.42\times{10}^{10}\geq0$  (21):1$.44\times{10}^{13}\geq0$  (23):$604100(n=3)\geq5\times{10}^{5}$  (24)$k=1:667991\geq5\times{10}^{5}$  (24)$k=2:667991\geq5\times{10}^{5}$ | Impossible; drug 2 is produced; (19) and (21) are always satisfied |
| 11 | Disease contraction with drug production and buying drug 1 | (11) is satisfied; (19) is satisfied when $j=1$, (19) is not satisfied when $j=2$; (25) or (26) is satisfied. | (11):$-160019\geq-160425$  (17):1$.44\times{10}^{13}\geq0$  (19)$g_{dj}=1,j=1:6.49\times{10}^{10}\geq0$  (19)$g_{dj}=1,j=2:1.42\times{10}^{10}\geq0$  (21):1$.44\times{10}^{13}\geq0$  (25)$j=1:640098\geq5\times{10}^{5}$  (25)$j=2:640058\geq5\times{10}^{5}$  (26)$j=k=1:703989\geq5\times{10}^{5}$  (26)$j=1,k=2:703989\geq5\times{10}^{5}$  (26)$j=2,k=1:703949\geq5\times{10}^{5}$  (26)$j=k=2:703949\geq5\times{10}^{5}$ | Impossible; drug 2 is produced; (19) and (21) are always satisfied |
| 12 | Disease contraction with drug production, but without buying drugs 1 or 2 | (11) and (12) are not satisfied; (19),(23),(24) are satisfied. | (11):$-160019\geq-160425$  (12):$-160425<-160019$  (17):1$.44\times{10}^{13}\geq0$  (19)$g_{dj}=1,j=1:6.49\times{10}^{10}\geq0$  (19)$g_{dj}=1,j=2:1.42\times{10}^{10}\geq0$  (21):1$.44\times{10}^{13}\geq0$  (23):$604100(n=3)\geq5\times{10}^{5}$  (24)$k=1:667991\geq5\times{10}^{5}$  (24)$k=2:667991\geq5\times{10}^{5}$ | Impossible; (11) is satisfied and hence person $i$ does buy drug 1 |
| 13 | Disease contraction with drug production and buying drug 2 | (12) and (19)  are satisfied;  (25) or (26) is satisfied. | (12):$-160425<-160019$  (17):1$.44\times{10}^{13}\geq0$  (19)$g_{dj}=1,j=1:6.49\times{10}^{10}\geq0$  (19)$g_{dj}=1,j=2:1.42\times{10}^{10}\geq0$  (21):1$.44\times{10}^{13}\geq0$  (25)$j=1:640098\geq5\times{10}^{5}$  (25)$j=2:640058\geq5\times{10}^{5}$  (26)$j=k=1:703989\geq5\times{10}^{5}$  (26)$j=1,k=2:703989\geq5\times{10}^{5}$  (26)$j=2,k=1:703949\geq5\times{10}^{5}$  (26)$j=k=2:703949\geq5\times{10}^{5}$ | Impossible; (12) is not satisfied and hence person $i$ does not buy drug 2 |
| 14 | Disease contraction with drug production and buying drug 1 | (11) and (19)  are satisfied;  (25) or (26) is satisfied. | (11):$-160019\geq-160425$  (17):1$.44\times{10}^{13}\geq0$  (19)$g_{dj}=1,j=1:6.49\times{10}^{10}\geq0$  (19)$g_{dj}=1,j=2:1.42\times{10}^{10}\geq0$  (21):1$.44\times{10}^{13}\geq0$  (25)$j=1:640098\geq5\times{10}^{5}$  (25)$j=2:640058\geq5\times{10}^{5}$  (26)$j=k=1:703989\geq5\times{10}^{5}$  (26)$j=1,k=2:703989\geq5\times{10}^{5}$  (26)$j=2,k=1:703949\geq5\times{10}^{5}$  (26)$j=k=2:703949\geq5\times{10}^{5}$ | Possible; (11) is satisfied and hence person $i$ does buy drug 1 |
